# Fluid balance, electrolyte, and neurohormonal responses to isotonic vs. hypotonic maintenance crystalloids in healthy Beagle dogs

**DOI:** 10.3389/fvets.2026.1948054

**Published:** 2026-10-09

**Authors:** Jiwoong Her, Daniel Gordon, Keon Kim, Jessica Wallinger, Catherine Langston

**Affiliations:** 1 Department of Clinical Sciences, College of Veterinary Medicine, North Carolina State University, Raleigh, NC, United States; 2 Veterinary Emergency Medicine, Department of Veterinary Clinical Science, College of Veterinary Medicine, Research Institute for Veterinary Science, Seoul National University, Seoul, Republic of Korea; 3 Department of Veterinary Clinical Sciences, College of Veterinary Medicine, The Ohio State University, Columbus, OH, United States; 4 Department of Nephrology and Extracorporeal Therapies, Colorado Animal Specialty and Emergency, Boulder, CO, United States; 5 Laboratory of Veterinary Internal Medicine, College of Veterinary Medicine and Veterinary Medical Research Institute, Jeju National University, Jeju, Republic of Korea

**Keywords:** crystalloid solutions, dog, fluid balance, hyponatremia, maintenance fluid therapy, tonicity, urinary sodium excretion

## Abstract

**Objective:**

To compare the effects of isotonic vs. hypotonic maintenance crystalloid therapy on fluid balance, urine output, and electrolyte homeostasis in healthy Beagles.

**Methods:**

In this 2-phase clinical study, nine healthy Beagles received intravenous isotonic (lactated Ringer's solution with 5% dextrose plus 26 mEq/L KCl) and hypotonic (Normosol-M and 5% dextrose plus 17 mEq/L KCl) crystalloids at an allometric maintenance rate for 48 h, with a 10-day washout between treatments from July 3 to September 29, 2021. Variables of interests were assessed every 12 h during treatment, and results were analyzed with baseline-adjusted linear mixed-effects models. A *p*-value < 0.05 was considered statistically significant. Timepoint-specific comparisons were exploratory and unadjusted for multiplicity.

**Results:**

In exploratory timepoint-specific analyses, calculated fluid balance was greater with isotonic crystalloids only at T36 [estimated marginal mean (EMM) difference, 356 ml (95% CI, 105–606)]. The overall treatment-by-time interaction for calculated fluid balance was not significant. Urine output did not differ, whereas cumulative urine output was higher with isotonic crystalloids only at T12 (back-transformed EMMs, 125.3 ml for isotonic vs. 81.2 ml for hypotonic). Isotonic crystalloids were associated with significantly higher urinary sodium and chloride at all time points than hypotonic crystalloids. Serum sodium was lower with hypotonic fluids at T24. Individual concentrations fell modestly below the lower reference limit during both phases, with a lowest recorded value of 137 mEq/L and a maximum interval-averaged rate of decline of 0.58 mEq/L/h, and recovered toward baseline by T48.

**Conclusions:**

In this exploratory study, isotonic maintenance crystalloids were associated with a transient, relatively more positive calculated fluid balance and consistently greater urinary sodium and chloride excretion than hypotonic crystalloids.

**Clinical relevance:**

In healthy Beagles, the two maintenance crystalloid formulations produced distinct patterns of renal sodium, chloride, and electrolyte-free water handling, while the hypotonic crystalloid did not cause a rapid or clinically important decline in serum sodium.

## Introduction

Intravenous fluid therapy is among the most common interventions in hospitalized dogs, and appropriate volume management requires understanding the underlying pathophysiology, assessing volume status, and selecting an appropriate solution (1, 2). The 2024 American Animal Hospital Association (AAHA) Fluid Therapy Guidelines for Dogs and Cats divide fluid therapy into resuscitation, rehydration, and maintenance phases (3). Human studies have shown that cumulative fluid exposure during hospitalization is often greatest during the maintenance phase (4).

Maintenance fluids are intended to provide water to meet physiologic losses and commonly classified by tonicity which is largely determined by Na^+^ concentration. The optimal choice between isotonic and hypotonic solutions for maintenance fluid therapy remains controversial. When isotonic crystalloids are administered at a maintenance rate, the amount of sodium and chloride provided may exceed basal requirements (5, 6). The salt load requires greater renal clearance, which can contribute to volume overload in critically ill patients (7). Conversely, pediatric studies have demonstrated an association between hypotonic fluids and an increased risk of hyponatremia in hospitalized children leading current pediatric guidelines to recommend isotonic solutions for patients aged 28 days to 18 years (8–12).

The Metabolism of Isotonic vs. Hypotonic Maintenance Solutions in Fasting Healthy Adults (MIHMoSA) trial (5), a crossover study comparing isotonic and hypotonic maintenance fluid therapy in fasting healthy adult volunteers, reported that isotonic crystalloids administered at maintenance rates are associated with increased fluid retention, hyperchloremia, and reduced urine output (5, 13–15). To the authors' knowledge, no controlled study in awake dogs has directly compared the physiologic responses to isotonic and hypotonic crystalloids during the maintenance phase, leaving current recommendations largely dependent on physiologic reasoning and extrapolation from human data (3, 12, 16, 17).

This study aimed to characterize fluid balance and physiological responses to isotonic (Lactated Ringer's solution (LRS) + 5 % dextrose, supplemented with 26 mEq/L of potassium chloride (KCl), Tonicity: 260 mOsm/L) and hypotonic crystalloids (Normosol-M and 5% dextrose, supplemented with 17 mEq/L of KCl, Tonicity: 80 mOsm/L) (Table 1) intravenously administered at an allometric fluid rate [132 x body weight (kg)^0.75^ / day] in healthy Beagles. Specifically, we aimed to evaluate fluid balance over 48 h by assessing net fluid balance (current body weight minus baseline body weight at each data point), and cumulative urine output from T0 to each predefined time point (T12, T24, T36, and T48). We also aimed to investigate the physiological mechanisms of osmoregulation and volume regulation by integrating measures of serum aldosterone and antidiuretic hormone (ADH) concentrations, serum osmolarity, urine osmolality, and serum and urine electrolytes. We hypothesized that a maintenance rate of isotonic crystalloids would result in a relatively more positive calculated fluid balance in healthy dogs than would hypotonic crystalloids.

**Table 1:** Composition of the isotonic and hypotonic crystalloids administered in a study of maintenance fluid therapy in 9 healthy Beagles.

Fluid	*Na*^+^ (mEq/L)	*K*^+^ (mEq/L)	*Cl*^−^(mEq/L)	Ca^2+^ (mEq/L)	Mg^2+^ (mEq/L)	*Lactate*^−^(mEq/L)	*Acetate*^−^(mEq/L)	Dextrose (g/L)	aSID	Osmolarity (mOsm/L)	Tonicity (mOsm/L)
Isotonic crystalloid	130	30	135	2.7	0	28	0	50	25	598	260
Hypotonic crystalloid	40	30	57	0	3	0	16	50	13	418	80

Nine healthy Beagles received both isotonic and hypotonic crystalloids in an experimental 2-phase clinical study, with two 48-h treatment phases separated by a 10-day washout. Each crystalloid was prepared by supplementing a commercial base fluid with potassium chloride (and, for the isotonic group, 5% dextrose); for the hypotonic bag, an equal volume was first removed to maintain a 1-L final volume. Potassium supplementation was selected to achieve the same final potassium concentration of 30 mEq/L in both prepared crystalloids. As the base potassium concentrations were 4 mEq/L in lactated Ringer's solution and 13 mEq/L in Normosol-M and 5% dextrose, 26 and 17 mEq of KCl were added to each 1-L bag, respectively. Isotonic crystalloid: lactated Ringer's solution (20) + 5% dextrose (21)+ 26 mEq/L potassium chloride (22). Hypotonic crystalloid: Normosol-M and 5% Dextrose (23) + 17 mEq/L potassium chloride (22). Cation and anion values are mEq/L. Calculated osmolarity (mOsm/L) = 2 × (*Na*^+^ + *K*^+^) + BUN (mg/dL)/2.8 + glucose (mg/dL)/18. Tonicity (mOsm/L) = 2 × (*Na*^+^). aSID (abbreviated strong ion difference) = *Na*^+^ + *K*^+^ – *Cl*^−^. Dextrose is presented in g/L; 50 g/L corresponds to 5,000 mg/dL, the unit used in the calculated-osmolarity equation.

## Materials and methods

This is an experimental, two-phase clinical study in nine healthy Beagles conducted between 3 July 2021 and 29 September 2021, the design of which was modeled after the MIHMoSA trial (5). The protocol was approved by The Ohio State University Institutional Animal Care and Use Committee (No. 2021A00000030). Dogs were obtained from the Laboratory Animal Resources Unit and housed at the Sisson Hall Vivarium and underwent a minimum 5-day acclimation period prior to the start of each experimental period. Each dog was individually housed in a stainless-steel run under standard vivarium conditions, with room temperature maintained at 22 ± 2 °C (72 ± 4 °F), relative humidity between 30% and 70%, in accordance with the Guide for the Care and Use of Laboratory Animals (18). A 12:12-h light:dark cycle was used. Dogs received a standard laboratory canine maintenance diet (LabDiet 5007 Canine Maintenance; PMI Nutrition International), which contains 0.38 % sodium (= 3,800 mg/kg dry matter) (19).

For inclusion, dogs were required to be considered healthy based on physical examination, complete blood count (CBC), serum biochemistry, and complete urinalysis (urine specific gravity, dipstick, and sediment examination) to rule out evidence of urinary tract infection. During each study period, heart rate, respiratory rate, and body temperature were monitored every 12 h.

The study consisted of two 48-h study periods, during which dogs received the isotonic crystalloid first and the hypotonic crystalloid during the second phase; the order of treatments was not randomized. The periods were separated by a 10-day washout to mitigate potential residual effects from the preceding treatment. The isotonic crystalloid used was lactated Ringer's solution (LRS, Fresenius Kabi) (20) with 5 % dextrose (Hospira) (21), supplemented with 26 mEq of potassium chloride (KCl, Hospira) (22) per 1 liter bag. The hypotonic crystalloid used was a balanced multiple-electrolyte maintenance solution (Normosol-M and 5% dextrose, ICU Medical) (23), supplemented with 17 mEq of KCl per 1 liter bag (Osmolarity: 418 mOsm/L; Tonicity: 80 mOsm/L). An equivalent volume of fluid was removed from the original 1-liter bag prior to supplementation to maintain a final volume of 1 liter. The composition of each fluid is outlined in Table 1. Fluids were infused (mL/h) with an electronic pump using the allometric fluid rate formula for dogs [132 x BW(kg)^0.75^/ 24 h] (3, 6). The electronic infusion pump (Sigma Spectrum IV infusion pump; Baxter Healthcare) used for this study was routinely calibrated for clinical use and was calibrated before use.

Beagles were sedated the day before the study period to avoid any impact from sedation, anxiety, or pain on the study outcomes, including potential effects of dexmedetomidine on urine output and ADH (24–26). A 5-Fr double lumen indwelling jugular venous catheter (MILACATH. MILA International) was placed to facilitate multiple blood samples. An indwelling urinary catheter (MILACATH. MILA International) was placed to empty the urinary bladder and measure urine output throughout the study period. In brief, a peripheral IV catheter was placed and then the dogs were sedated with butorphanol (Torbugesic, Zoetis) (0.2 mg/kg IV) and dexmedetomidine (Dexdomitor, Zoetis) (5–10 μg/kg IV) to aid in catheter placement and were reversed with a dose of atipamezole (Antisedan, Zoetis) (0.1 mg/kg IM) once the procedure was completed. An Elizabethan collar was placed on each dog. Dogs were then fasted with water and food removed starting at 12AM until the morning of the study when fluids were administered at 8 AM. All oral intake was discontinued for the rest of the 48 h study period and resumed immediately upon completion of the study period. Three investigators (JH, DG, JW) constantly monitored the dogs for complications associated with catheterization and fluid therapy.

Throughout the study period, blood (3 ml) was collected from the indwelling jugular catheter and urine collected from the urinary drainage bag at baseline (T0) and every 12 h (T12, T24, T36, T48) thereafter. Blood samples were collected from the central venous catheter using a modified three-syringe technique described previously (27). Blood was collected in an ethylenediaminetetraacetic acid (EDTA) tube and submitted to The Ohio State University Veterinary Medical Center (OSU-VMC) Clinical Pathology Laboratory for CBC. Serum samples were collected in a serum-separator tube, allowed to clot at room temperature for 30 min before centrifugation at 3,000 × g for 15 min at 4 °C, and then submitted to the same laboratory for serum chemistry. For serum aldosterone analysis, 1–2 ml of serum were aliquoted and transferred to a −80 °C freezer immediately after collection and stored until the measurement. For ADH measurement, additional precautions were taken to minimize peptide degradation. Aliquot of each whole blood sample were placed into pre-chilled EDTA tubes prefilled with a combination of protease inhibitors (D-phenylalanine-arginine chloromethyl ketone, benzamidine, aprotinin, leupeptin hemisulfate, and DL-thiorphan). The tubes were immediately centrifuged at 4 °C (1,600 × g for 15 min) and then immediately placed on ice within 10 min of collection. Plasma samples were then transferred to polyethylene tubes and stored at −80 °C until analysis.

Urine output was measured by emptying the urine collection bags every 6 to 12 h to ensure accurate measurement of urine output and cumulative fluid balance. Body weight was measured every 12 h using the same scale (The VSSI way, Kanawha Scales & Systems) which is routinely calibrated for experimental uses.

The primary endpoints were calculated fluid balance, cumulative urine output (ml), and urine output (ml/kg/h). These endpoints were evaluated at each predefined postbaseline time point (T12, T24, T36, and T48). Calculated fluid balance was defined as body weight at each time point minus baseline body weight and was expressed as volume. Cumulative urine output (ml) was calculated from T0 to each time point, and urine output (ml/kg/h) was calculated for each 12-h interval and normalized to body weight. Secondary endpoints were markers of osmoregulation (calculated serum osmolarity mOsm/L, serum ADH concentrations pg/ml) and volume regulation (serum aldosterone, urine sodium, urine measured osmolality and calculated osmolarity, and serum albumin). In addition, clinically important electrolytes (sodium, potassium, chloride and calcium) were compared between the isotonic and hypotonic groups. Serum chemistry, urine glucose, and urine electrolyte were quantified by using commercial blood analyzer (Cobas c 311 analyzer; Roche Diagnostics). Abbreviated strong ion difference (aSID) was calculated as [*Na*^+^] + [K^+^]– [*Cl*^−^] (mEq/L) (28). Serum aldosterone concentrations were measured by radioimmunoassay kit (ACTIVE Aldosterone RIA; Beckman Coulter Inc) at the Michigan State University Veterinary Diagnostic Laboratory. Plasma ADH concentrations were measured using a canine ADH ELISA kit (Canine Anti-Diuretic Hormone ADH ELISA Kit; Enzo Life Sciences) according to the manufacturer's instructions (29).

Calculated osmolarity (mOsm/L) of crystalloid, serum, and urine in this manuscript was derived using the following equation: calculated osmolarity (mOsm/L) = 2 x (Na^+^+K^+^) [mEq/L] + BUN (mg/dL)/2.8 + Glucose (mg/dL)/18 (30). Tonicity (mOsm/L) was calculated using the following equation: 2 (Na+) [mEq/L], with glucose considered an ineffective osmole *in vivo* (30, 31). Urine osmolality (mOsm/kg) was measured using a freezing point depression osmometer (Advanced Instruments Osmometer, Model 3320; Nova Biomedical) at the Cornell University Animal Health Diagnostic Center.

### Statistical analysis

Sample size was determined *a priori* using G^*^Power (version 3.1.9.7; Heinrich-Heine-Universität Düsseldorf). Based on data from the MIHMoSA trial (5), a clinically relevant between-condition difference in urine output of 5 mL/kg was prespecified. For a matched-pairs comparison, this corresponded to a standardized effect size (dz) of approximately 1.2. With a two-sided α of 0.05 and 80% power, eight dogs were required. To allow for potential incomplete data or dropout, nine dogs were enrolled.

All analyses were performed using SAS, version 9.4 (SAS Institute Inc), statistical significance set at *p* < 0.05. Repeated measurements were analyzed using linear mixed-effects models. Each model included fixed effects for treatment (isotonic vs. hypotonic), time, and the treatment-by-time interaction, with a random intercept for each dog to account for within-subject correlation.

To ensure that treatment comparisons reflected differences attributable to the assigned fluid type rather than baseline imbalance, a baseline value of the corresponding endpoint was included as a covariate when a non-zero baseline was present, so that treatment effects represent baseline-adjusted differences. Because each dog received both treatments and baseline was modeled as a covariate rather than a separate time point, between-treatment comparisons reflect intervention effects rather than baseline imbalance.

Model assumptions were assessed using histograms and quantile–quantile plots (Q–Q plots) of conditional residuals (normality) and plots of conditional residuals vs. fitted values (homogeneity of variance). When necessary, outcomes were log-transformed to satisfy model assumptions. Linear mixed-effects models were fitted using restricted maximum likelihood estimation. Treatment effects at each time point were evaluated using model-based estimated marginal means (EMM), and degrees of freedom were calculated using the Satterthwaite approximation. To allow direct comparison of baseline (T0 values), alternative linear models with the same model structures except with the baseline covariate removed and T0 data included as an additional timepoint were used. Outcomes that were log-transformed for the primary analysis were also log-transformed for baseline comparison.

Descriptive data are presented as mean ± standard deviation (SD) to remain consistent with the linear mixed model analytic approach. For each endpoint, between-treatment effect sizes are reported as the model-based EMM difference (isotonic – hypotonic) with 95% confidence intervals (CI) and *p* values. For log-transformed endpoints, results are presented as back-transformed EMMs (geometric means) for each treatment with 95% CIs, and the corresponding fold change (ratio of back-transformed EMMs) is reported as a standardized effect size, because such variables cannot be subtracted on the original scale.

Twelve-hour urine output values were missing for 2 dogs during the hypotonic crystalloid phase due to technical issues with urine collection at T12. The mean value of the other 7 measured animals at 12 h was used to estimate cumulative urine output for the missing animals. The imputed value propagated into each dog's cumulative urine output, including the 48-h total; a sensitivity analysis excluding these 2 dogs (*n* = 7) addressed this.

Two sensitivity analyses were performed. First, because 12-h urine output values were missing for 2 dogs and were imputed using the group mean, the cumulative urine output model was refitted excluding these 2 dogs to confirm that the imputation did not materially affect the results. Second, for serum chloride, abbreviated strong ion difference and urine glucose, the models were refitted after excluding values visually identified as extreme by examining line plots for each animal and period separately, and Wilcoxon signed-rank tests were performed on both the absolute values and the change-from-baseline values of these endpoints to confirm robustness of findings.

No adjustments for multiple statistical tests performed on multiple endpoints and timepoints were performed, so analyses should be considered exploratory.

In addition, overall (Type 3) tests of the treatment, time, and treatment-by-time interaction effects were additionally examined for each endpoint from the same linear mixed-effects models, and all timepoint-specific comparisons are presented as exploratory analyses (Table 2, Supplementary Table S3). For the variables whose baseline values differed between phases (plasma ADH, serum aldosterone, serum creatinine, urine chloride, and calculated serum osmolarity), sensitivity analyses were performed on the change from each animal's own phase baseline, using the same model structure, with paired Wilcoxon signed-rank tests at each time point (Supplementary Table S4).

**Table 2:** Overall (Type 3) tests of fixed effects and exploratory baseline-adjusted timepoint-specific comparisons (isotonic – hypotonic) of key fluid-balance, volume-regulatory, osmoregulatory, and electrolyte variables over time in 9 healthy Beagles described in Table 1.

Variable	Overall p value			Between-group difference at each time point (exploratory)			
	Treatment	Time	Treatment × time	T12	T24	T36	T48
Fluid balance and urine output							
Calc. fluid balance (ml)	0.081	**< 0.001**	0.17	33	61	**356** ^*^	−6
*p* value				0.791	0.627	**0.006**	0.965
Body weight (kg)	0.12	**< 0.001**	0.16	0.0	0.0	**0.3** ^*^	0.0
*p* value				0.863	0.693	**0.008**	0.890
Cumulative urine output^a^	**0.028**	**< 0.001**	0.41	**1.54** ^*^	1.10	1.14	1.09
*p* value				**0.012**	0.572	0.446	0.612
Urine output (mL/kg/h)^a^	0.12	**< 0.001**	0.53	1.57	0.99	1.13	1.13
*p* value				0.067	0.947	0.581	0.580
Volume regulation and osmoregulation							
Aldosterone (pmol/L)^a^	**0.035**	0.068	0.18	1.07	**0.46** ^*^	0.80	**0.41** ^*^
*p* value				0.845	**0.041**	0.547	**0.019**
ADH (pg/ml)	**< 0.001**	**0.042**	0.67	—	**−3.8** ^*^	—	**−3.4** ^*^
*p* value				—	**< 0.001**	—	**< 0.001**
Calculated urine osmolarity (mOsm/L)^a^	**< 0.001**	**< 0.001**	0.10	0.99	**1.52** ^*^	**1.53** ^*^	**1.40** ^*^
*p* value				0.918	**0.002**	**0.002**	**0.011**
Measured urine osmolality (mOsm/kg)^a^	0.056	**< 0.001**	0.49	0.94	1.36	1.42	1.48
*p* value				0.773	0.163	0.116	0.079
Calculated serum osmolarity (mOsm/L)	**0.013**	**0.004**	0.76	1.1	**4.5** ^*^	2.7	2.6
*p* value				0.546	**0.013**	0.126	0.142
Cumulative EFWC (ml)	**< 0.001**	0.49	**< 0.001**	−83	**−275** ^*^	**−444** ^*^	**−599** ^*^
*p* value				0.057	**< 0.001**	**< 0.001**	**< 0.001**
Serum and urine electrolytes							
Serum sodium (mEq/L)	**< 0.001**	**0.035**	0.33	1.7	**3.3** ^*^	1.4	1.3
*p* value				0.056	**0.001**	0.121	0.152
Serum chloride (mEq/L)	**0.001**	**0.042**	0.46	**2.5** ^*^	**2.9** ^*^	1.6	0.6
*p* value				**0.026**	**0.012**	0.150	0.604
Urine sodium (mEq/L)	**< 0.001**	**< 0.001**	0.073	**113** ^*^	**117** ^*^	**93** ^*^	**71** ^*^
*p* value				**< 0.001**	**< 0.001**	**< 0.001**	**< 0.001**
Urine chloride (mEq/L)	**< 0.001**	**< 0.001**	0.69	**79** ^*^	**106** ^*^	**104** ^*^	**89** ^*^
*p* value				**0.003**	**< 0.001**	**< 0.001**	**0.001**
Cumulative sodium balance (mEq)^b^	**< 0.001**	0.76	0.19	—	**31.9** ^*^	**28.0** ^*^	**51.5** ^*^
*p* value				—	**0.001**	**0.011**	**< 0.001**
Cumulative chloride balance (mEq)^b^	**< 0.001**	0.56	0.52	—	**36.7** ^*^	**35.0** ^*^	**52.2** ^*^
*p* value				—	**0.001**	**0.010**	**< 0.001**
FENa (%)^a^	**< 0.001**	**< 0.001**	0.32	**2.13** ^*^	**2.64** ^*^	**2.76** ^*^	**1.71** ^*^
*p* value				**< 0.001**	**< 0.001**	**< 0.001**	**0.010**

^**a**^Isotonic: hypotonic fold change [ratio of back-transformed geometric-mean estimated marginal means (i.e. geometric means)]; all variables without a superscript are the EMM difference (isotonic – hypotonic) on the original scale. ^*^Significant between-group difference (*p* < 0.05); significant values are shown in bold. *p* values are reported without a leading zero; *p* < 0.001 is reported as “ < 0.001.” ADH was compared only at T24 and T48. EFWC, electrolyte-free water clearance; FENa, fractional excretion of sodium; ADH, antidiuretic hormone; EMM, estimated marginal mean; CI, confidence interval. Per-group descriptive statistics (mean ± SD) and 95% CIs for every variable are provided in Supplementary Table S1. Overall *p* values are Type 3 tests of the treatment, time, and treatment-by-time interaction fixed effects from the same linear mixed-effects models; complete overall tests for all endpoints are provided in Supplementary Table S3, and timepoint-specific comparisons are exploratory and unadjusted for multiplicity. b Cumulative sodium or chloride balance = cumulative amount administered minus cumulative urinary excretion (mEq), analyzed from T24 onward (—, not calculable at T12); values are EMM differences (isotonic minus hypotonic).

Missing urine-output data were handled in three ways: the primary analysis using group-mean imputation, a prespecified sensitivity analysis excluding the 2 affected dogs (*n* = 7), and an additional analysis in which the missing values were left missing and handled directly by restricted maximum likelihood estimation (Supplementary Table S4).

For comparison with the MIHMoSA trial, fluid balance was also calculated as cumulative infused fluid volume minus cumulative urine output and analyzed as a supplementary endpoint. This calculation was not available at T12 of the isotonic phase, and intervals with imputed urine volumes were excluded (Supplementary Table S4).

Individual serum sodium concentrations were additionally examined descriptively. For each dog and each treatment, we calculated the change over every 12-h sampling interval, the largest single-interval decline, and the largest decline from that animal's own baseline. Because sodium was measured every 12 h, these values represent interval-averaged rates of change and cannot exclude more rapid, transient fluctuations between sampling points. No safety threshold for the rate of decline in serum sodium has been validated in dogs; a decline of more than 6 mEq/L over 12 h (an interval-averaged rate of >0.5 mEq/L/h) is reported for descriptive purposes only.

## Results

Nine healthy male intact Beagles aged 1–3 years, mean body weight, 12.6 ± 1.2 kg, were included. No dog had clinically relevant abnormalities on baseline screening, and vital parameters remained within reference ranges throughout the study.

The data pertaining to the primary objectives are summarized in Table 2. Group descriptive statistics (mean ± SD), baseline comparison, and the baseline-adjusted between-group differences (with 95% CIs and *p* values) for all measured and calculated variables are presented in the consolidated Supplementary Table 1. Most variables were comparable at baseline. However, plasma ADH (*p* < 0.001), serum aldosterone (*p* = 0.02), serum creatinine (*p* < 0.001), urine chloride (*p* = 0.01), and calculated serum osmolarity (*p* = 0.048) differed significantly, each being higher before the second (hypotonic) phase.

Calculated fluid balance differed between treatments only at T36, where it was relatively more positive with isotonic than hypotonic crystalloids; at that time point it was closer to baseline in the isotonic group and lower than baseline in the hypotonic group, and body weight showed a corresponding difference (Table 2). Differences at other time points were not significant, and by T48 both treatments had returned toward baseline (Figure 1). In the overall tests, neither the treatment effect (*p* = 0.081) nor the treatment-by-time interaction (*p* = 0.17) for calculated fluid balance was significant (Table 2). When fluid balance was instead calculated as cumulative infused volume minus cumulative urine output, following the approach in the MIHMoSA trial, no time point differed significantly between treatments (T36 estimated difference, −185 ml; 95% CI, −397 to 27; *p* =0.086; Supplementary Table S4). Urine output (ml/kg/h) did not differ at any time point (Table 2, Figure 1). In an exploratory time point-specific analysis, cumulative urine output was significantly higher with isotonic crystalloid at T12 and showed a higher pattern thereafter without reaching significance at T24–T48; excluding the 2 dogs with imputed 12-h values reproduced this pattern (significant only at T12) (Table 2, Figure 1). Leaving the missing 12-h values unimputed and relying on restricted maximum likelihood estimation provided nearly identical results (T12 fold change, 1.58; p =0.014; Supplementary Table S4).

**Figure 1:**
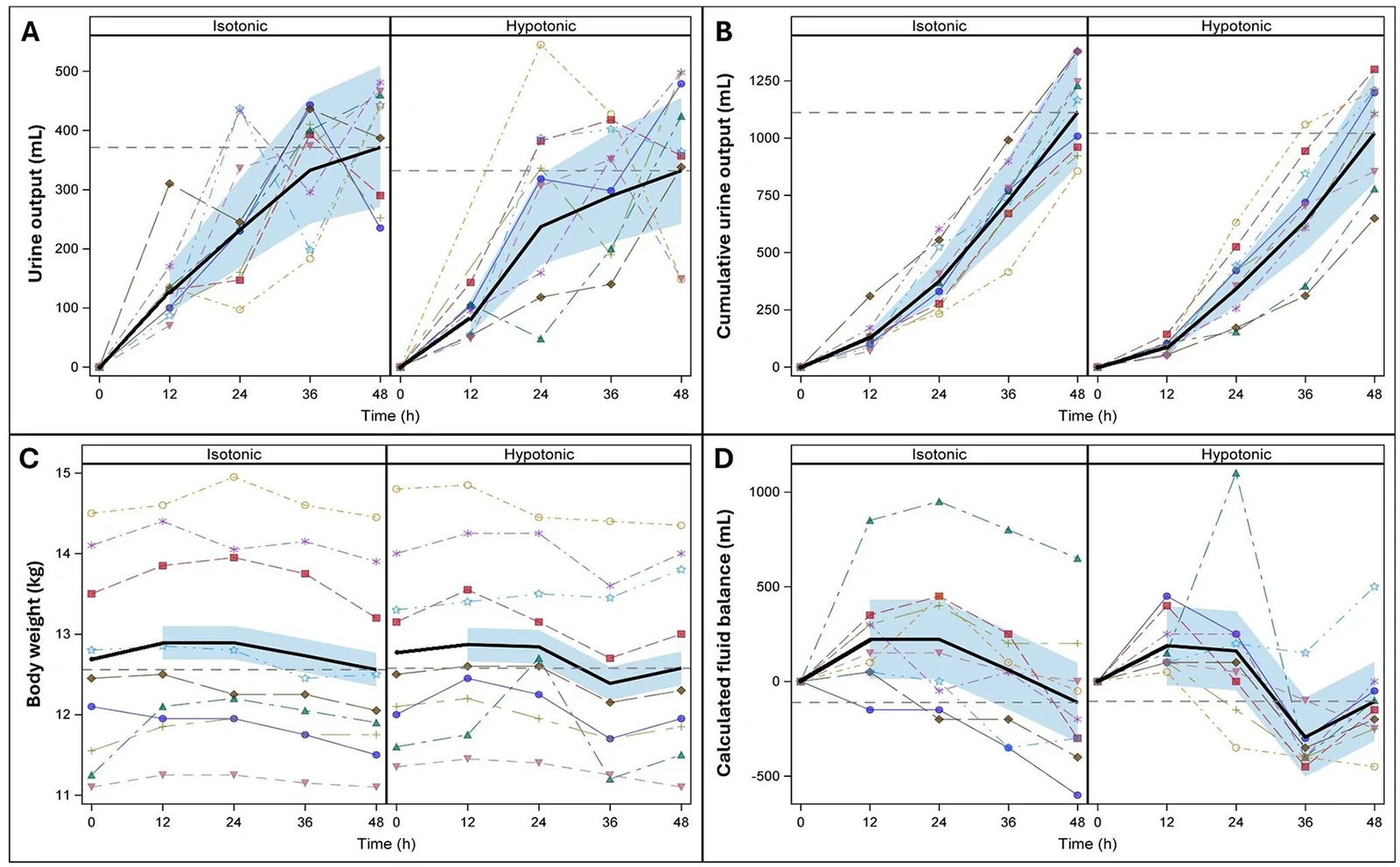
Time-dependent changes in **(A)** urine output, **(B)** cumulative urine output, **(C)** body weight, and **(D)** calculated fluid balance in 9 healthy Beagles that received isotonic (lactated Ringer's solution with 5% dextrose plus 26 mEq/L KCl) and hypotonic (Normosol-M and 5% dextrose plus 17 mEq/L KCl) crystalloids at an allometric maintenance rate for two 48-h periods separated by a 10-day washout. Within each panel, the left facet corresponds to isotonic crystalloid and the right facet to hypotonic crystalloid. Thin colored lines represent individual dogs; the bold black line is the marginal mean estimated from a linear mixed-effects model, and the shaded band is the 95% confidence interval. The horizontal dashed line in each facet indicates the model-predicted marginal mean at 48 h (t48). Calculated fluid balance was defined as body weight at each time point minus baseline (t0) body weight and was expressed as volume (1 kg = 1,000 ml). Timepoint-specific comparisons for urine output and cumulative urine output were considered exploratory as 12-h urine output values were missing for 2 dogs and no adjustment was made for multiple testing.

Serum aldosterone was lower in the isotonic group at T24 and T48, and plasma ADH (compared at T24 and T48) was significantly lower in the isotonic group at both times (Figure 2). However, in the change-from-baseline sensitivity analysis, neither hormone differed between treatments at any time point (overall treatment effect, *p* = 0.92 for ADH and *p* = 0.93 for aldosterone; Supplementary Table S4), whereas the treatment differences in urine chloride and calculated serum osmolarity persisted. Calculated urine osmolarity was higher in the isotonic group at T24–T48, whereas measured urine osmolality did not differ (Figure 2). Urine sodium and chloride were significantly higher, and urine potassium significantly lower (only at T12), in the isotonic group (Figure 3). Calculated serum osmolarity was higher in the isotonic group at T24, serum BUN was lower at T12 and T36, and serum albumin did not differ (Table 2).

**Figure 2:**
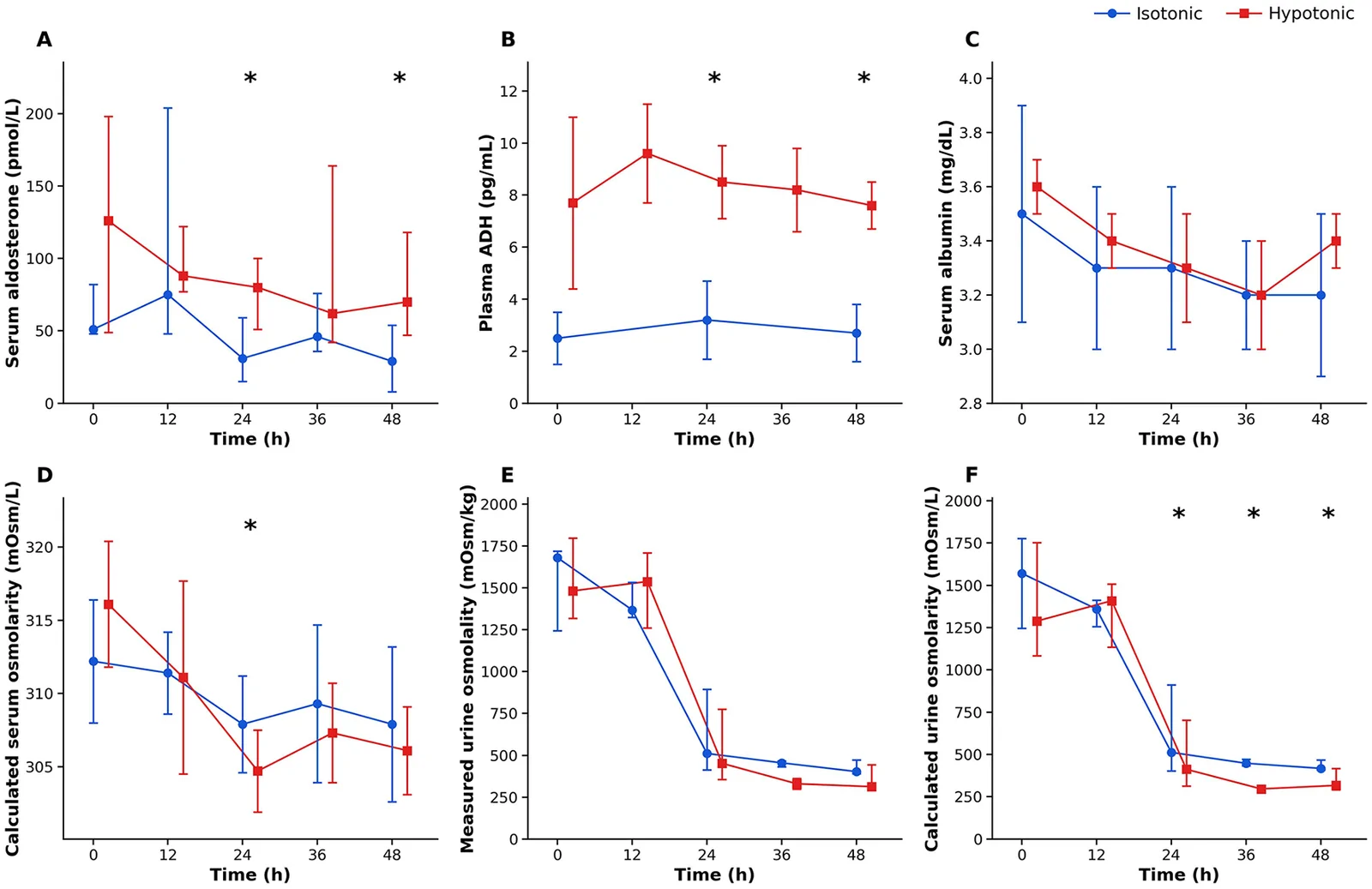
Time-dependent changes in volume-regulatory and osmoregulatory variables during isotonic (blue) and hypotonic (red) maintenance crystalloid administration in the 9 healthy Beagles described in Figure 1. **(A)** serum aldosterone (pmol/L), **(B)** plasma antidiuretic hormone (ADH, pg/ml), **(C)** serum albumin (mg/dL), **(D)** calculated serum osmolarity (mOsm/L), **(E)** measured urine osmolality (mOsm/kg), and **(F)** calculated urine osmolarity (mOsm/L). For each time point, symbols represent the group summary and whiskers the associated dispersion: panels A, E, and F (log-transformed variables) show the median and interquartile range (25th−75th percentile), whereas panels B, C, and D show the mean ± 1 SD. Isotonic and hypotonic symbols are horizontally offset to reduce overlap. Asterisks (*) indicate a significant between-group difference at that time point (*p* < 0.05).

**Figure 3:**
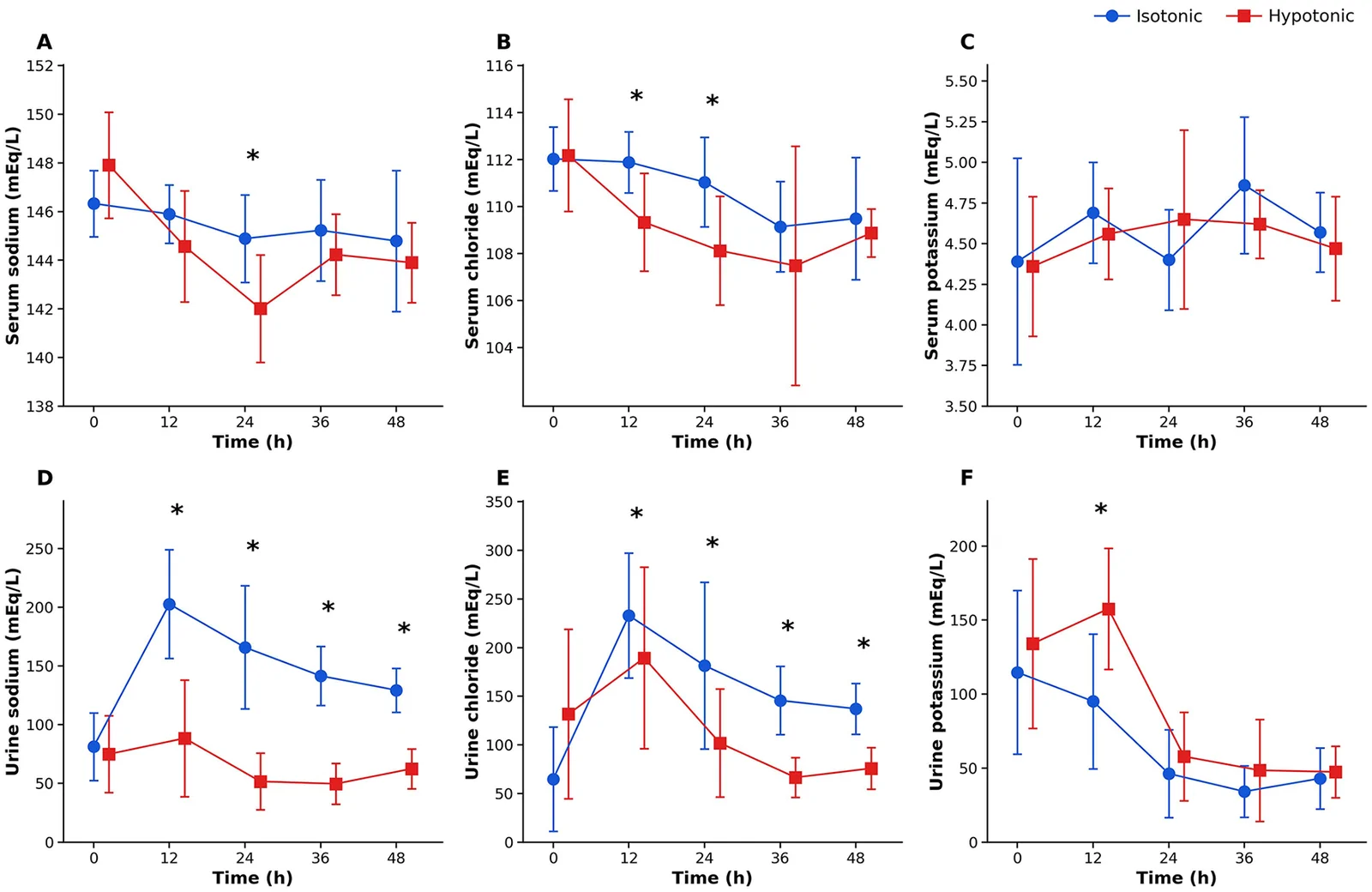
Time-dependent changes in serum and urine electrolyte concentrations during isotonic (blue) and hypotonic (red) maintenance crystalloid administration in the 9 healthy Beagles described in Figure 1. Serum electrolytes: **(A)** serum sodium (mEq/L), **(B)** serum chloride (mEq/L), and **(C)** serum potassium (mEq/L). Urine electrolytes: **(D)** urine sodium (mEq/L), **(E)** urine chloride (mEq/L), and **(F)** urine potassium (mEq/L). For each time point, symbols represent the group mean and whiskers represent ± 1 SD; isotonic and hypotonic symbols are horizontally offset to reduce overlap. Asterisks (*) indicate a significant between-group difference at that time point (*p* < 0.05). SD, standard deviation.

Serum sodium was lower in the hypotonic group, reaching significance at T24, and serum chloride was higher in the isotonic group at T12 and T24. Individual serum sodium concentrations fell below the lower reference limit (145-154 mEq/L) in both phases, whereas most other analytes remained within their reference intervals (Figure 3, Figure 4, Supplementary Table S1). Serum potassium, phosphate, and the abbreviated strong ion difference did not differ significantly. Serum calcium was lower in the isotonic group at T12 but remained within the reference interval. Serum and urine glucose and urine BUN did not differ between treatments, except lower urine glucose in the isotonic group at T24.

**Figure 4:**
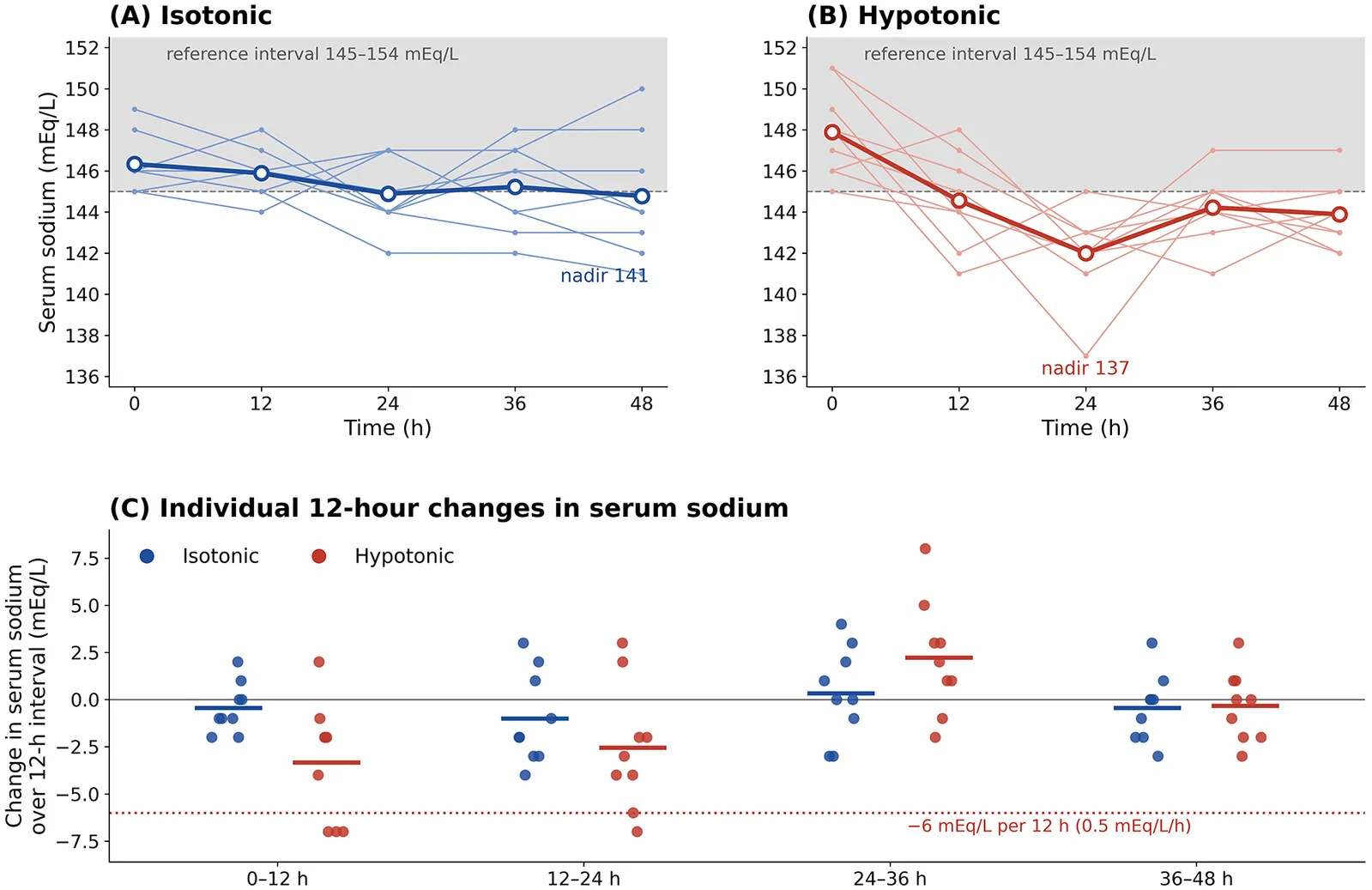
Individual serum sodium concentrations in 9 healthy Beagles that received isotonic (lactated Ringer's solution with 5% dextrose plus 26 mEq/L KCl) and hypotonic (Normosol-M and 5% dextrose plus 17 mEq/L KCl) crystalloids at an allometric maintenance rate for two 48-h periods separated by a 10-day washout. **(A)** Individual serum sodium trajectories during the isotonic phase. **(B)** Individual serum sodium trajectories during the hypotonic phase. In panels A and B, thin lines represent individual dogs, and the bold line with open circles represents the group mean; the shaded band indicates the laboratory reference interval (145–154 mEq/L), the dashed line its lower limit, and the lowest single concentration recorded (nadir) is annotated in each panel (141 mEq/L, isotonic; 137 mEq/L, hypotonic, in 1 dog at T24). **(C)** Individual changes in serum sodium over each 12-h sampling interval. Each symbol represents one dog (blue, isotonic; red, hypotonic), horizontal bars indicate group means, and negative values indicate a decrease. The dotted line marks a decline of 6 mEq/L per 12 h, corresponding to an interval-averaged rate of 0.5 mEq/L/h; this threshold has not been validated in dogs and is shown for descriptive purposes only.

Individual serum sodium trajectories are shown in Figure 4 and individual values in Supplementary Table S2. During the isotonic phase, the largest decline observed in any dog over a single 12-h interval was 4 mEq/L, the largest decline from an individual baseline was 5 mEq/L, and the lowest single concentration was 141 mEq/L. During the hypotonic phase, the largest single-interval decline was 7 mEq/L (interval-averaged, 0.58 mEq/L/h; observed in 4 of 9 dogs), the largest decline from an individual baseline was 9 mEq/L, and the lowest single concentration was 137 mEq/L (1 dog at T24). Concentrations below the lower reference limit (145 mEq/L) occurred in both phases (isotonic, 15 of 36 postbaseline values in 8 of 9 dogs. Hypotonic, 24 of 36 values in all 9 dogs). In the hypotonic phase the group mean reached its nadir at T24 (142 mEq/L) and recovered toward baseline by T36-T48, whereas isotonic group means changed little throughout (145-146 mEq/L). No dog developed a concentration below 137 mEq/L or showed clinical signs attributable to hyponatremia.

Cumulative electrolyte-free water clearance (EFWC) became progressively more negative in the isotonic group than in the hypotonic group across T24–T48, and fractional excretion of sodium (FENa) was significantly higher in the isotonic group at all time points (Table 2, Figure 5). Cumulative sodium and chloride balance (intake minus urinary excretion) was more positive with the isotonic crystalloid from T24 onward (at T48: sodium, +51 mEq; chloride, +52 mEq; both *p* < 0.001; Table 2 and Supplementary Table S4).

**Figure 5:**
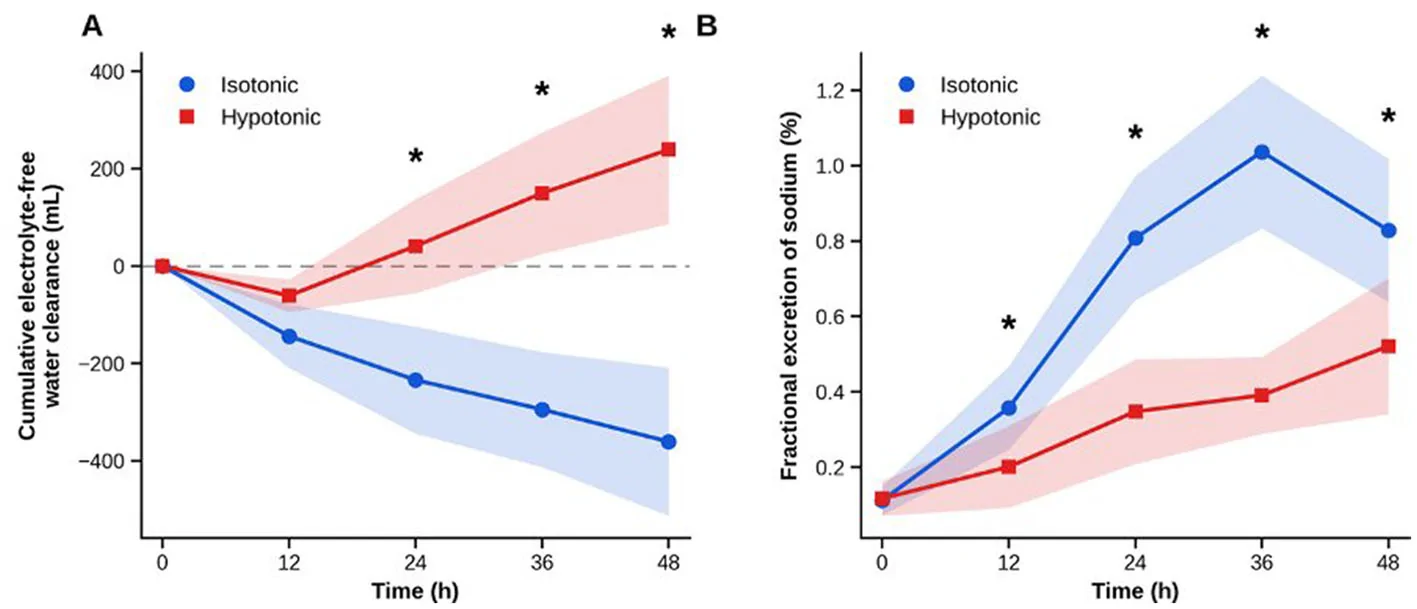
Time-dependent changes in **(A)** cumulative electrolyte-free water clearance (ml) and **(B)** fractional excretion of sodium (%) during isotonic (blue) and hypotonic (red) maintenance crystalloid administration in the 9 healthy Beagles described in Figure 1. Solid lines represent the group mean and shaded bands the 95% confidence interval; isotonic data are shown as circles and hypotonic data as squares. In panel A, the dashed horizontal line denotes zero clearance: positive values indicate net electrolyte-free water excretion and negative values indicate net electrolyte-free water retention. Asterisks (*) indicate a significant between-group difference at that time point (*p* < 0.05). EFWC, electrolyte-free water clearance; FENa, fractional excretion of sodium. Electrolyte-free water clearance (EFWC, ml = urine volume × [1 – (urine Na + urine K) ÷ serum Na]). Fractional excretion of sodium (FENa, % = (urine Na × serum creatinine) ÷ (serum Na × urine creatinine.) × 100)

## Discussion

In the present study, isotonic and hypotonic crystalloids produced distinct types of fluid and electrolyte handling when administered at an allometric maintenance rate. The most consistent differences between the two crystalloids were in renal solute handling, including urinary sodium and chloride excretion, FENa, cumulative sodium and chloride balance, and electrolyte-free water clearance, rather than in fluid balance itself. The isotonic crystalloid group was associated with a more positive calculated fluid balance that reached statistical significance at T36 only in exploratory timepoint-specific comparisons, without a significant overall treatment-by-time interaction. In addition, the isotonic crystalloid group had higher urinary sodium and chloride excretion along with higher FENa at all points. Cumulative EFWC was correspondingly more negative in the isotonic group at T24-T48, indicating net retention of electrolyte-free water. The fluid-balance pattern was more modest than that reported in the MIHMoSA trial (5).

Several similarities and differences should be considered before direct comparison between the present study and the MIHMoSA trial (5). Both studies used a two-period crossover design with 48-hour periods with maintenance-rate infusions in fasted subjects; however, MIHMoSA balanced the treatment sequence across matched pairs of participants (5), whereas in the present study all dogs received the isotonic crystalloid first. The present study used an allometric maintenance rate, whereas the human study used weight-based maintenance rates of 25 ml/kg. The MIHMoSA study (5) compared a sodium chloride–based isotonic solution (0.9% NaCl in 5% glucose with 40 mmol/L KCl) with a premixed hypotonic solution (0.32% NaCl, 54 mmol/L sodium and 55 mmol/L chloride). The isotonic fluid chosen in this study was a balanced crystalloid with less sodium compared to the fluid used in MIHMoSA trial (5). These differences in fluid composition, together with species differences and calculation of maintenance fluid rates, should be considered while interpreting these two studies. Furthermore, the two study solutions differed not only in tonicity but also in sodium, chloride, calcium, magnesium, lactate, acetate, and buffer content. The comparison is therefore between two crystalloid formulations classified by their tonicity, and no single constituent, including tonicity itself, can be isolated as the cause of any particular difference observed. The species differences include dogs' substantially lower basal aldosterone concentrations, lower obligatory sodium requirements, allometric rather than linear maintenance fluid scaling, and differences in renal concentrating capacity and the osmotic threshold for ADH release (30, 32). More importantly, the studies also differed in how fluid balance was assessed: MIHMoSA trial (5) calculated it as infused fluid volume minus cumulative urine output, whereas here it was defined as each dog's body weight at each time point minus its baseline body weight. We chose this method to capture insensible losses and to reflect the most common veterinary practice of using body weight change as a surrogate of hydration status.

The study was designed as a within-subject crossover, following the MIHMoSA trial (5), so that each dog served as its own control. Unlike MIHMoSA(5), which balanced treatment sequence between matched participants, the present study used a homogeneous cohort of purpose-bred Beagles of the same sex and breed, with similar age and body weight, maintained under standardized conditions. Given this homogeneity and practical constraints, a fixed treatment sequence was used. However, this design cannot fully distinguish treatment effects from period or order effects. Baseline plasma ADH, serum aldosterone, serum creatinine, urine chloride, and calculated serum osmolarity differed significantly between phases. Although a 10-day washout and baseline-adjusted analyses were used to reduce these effects, residual order effects cannot be excluded; therefore, findings for aldosterone and ADH should be interpreted with caution.

In the present study, calculated fluid balance differed between treatments only at T36, and its baseline-relative pattern differed from that reported in the MIHMoSA trial (5). Whereas MIHMoSA reported a progressive, sustained increase in the isotonic group, here fluid balance rose transiently at T12–T24 in both treatments and, at T36, was closer to baseline with isotonic and below baseline with hypotonic crystalloid (61 vs. −294 ml) before both returned toward baseline by T48 (Figure 1). These findings should therefore be interpreted as a modest relative between-treatment difference under controlled conditions in healthy Beagles rather than an absolute or sustained increase in fluid retention. Several factors may account for this. The isotonic crystalloid was less sodium-rich than the 0.9% NaCl used in the human trial and likely provided a weaker stimulus for volume expansion. In addition, calculated fluid balance was derived from body-weight change, which incorporates unmeasured insensible and fecal losses (e.g., respiratory water loss through panting); because the same fasting and housing conditions applied to both arms, these would have affected both similarly, again supporting a relative rather than absolute interpretation.

In contrast to the MIHMoSA trial (5), in which isotonic fluid was associated with lower urine output and greater retention, cumulative urine output was higher with the isotonic crystalloid at T12 (Figure 1) while total urine volume did not differ between treatments. This appeared to reflect a divergent ADH response (33, 34). ADH was significantly lower with isotonic treatment (T24 and T48), whereas it did not differ between fluids in MIHMoSA (5), together with a concurrent natriuretic diuresis. We postulate that the sodium-driven tendency toward volume retention with the isotonic crystalloid was partly offset by this solute diuresis, so that the lower aldosterone and ADH reflect a transiently expanded effective circulating volume rather than driving the diuresis. This interpretation is supported by the higher urinary sodium, chloride, and FENa accompanying the larger urine volume, and by a progressively more negative cumulative EFWC, indicating net conservation of electrolyte-free water despite the higher urine volume (Figure 5). Serum and urine creatinine were within reference limits and comparable between phases, consistent with the higher FENa reflecting differences in renal sodium handling. Treatment-related differences in renal sodium handling and FENa have likewise been reported with crystalloid fluid therapy in horses (35). Together these findings indicate comparable diuretic volumes but greater obligate sodium and chloride excretion, a difference in urine composition rather than volume. Further studies are warranted to investigate these mechanisms in healthy and critically ill dogs.

A relatively more positive calculated fluid balance with the isotonic crystalloid would also be consistent with the lower serum aldosterone at T24 and T48 along with lower serum BUN, higher urine sodium, chloride at all time points. This is similar to the MIHMoSA trial in which an isotonic group showed significantly lower serum aldosterone. Despite dogs' relatively low circulating aldosterone compared with humans, statistically significant between-group difference was nonetheless detected, supporting the robustness of this finding. However, baseline serum aldosterone was also significantly higher before the hypotonic phase (*p* = 0.02), limiting interpretation of the effect of fluid type on serum aldosterone. When aldosterone was analyzed as change from each phase's own baseline, no between-treatment difference remained at any time point (Supplementary Table S4). Calculated urine osmolarity was higher in the isotonic group between T24 and T48, reflecting the significantly higher urinary sodium excretion, whereas measured urine osmolality showed a positive pattern that did not reach statistical significance. This may reflect the small sample size and the contribution of unmeasured non-sodium osmoles that are detected by osmometer but not by the calculated estimate. Measured urine osmolality, however, did not differ significantly between treatments. Thus, conclusions about renal water handling, which rely mainly on the calculated values, should therefore be interpreted cautiously. In contrast to the MIHMoSA trial (5), in which significantly lower serum albumin in the isotonic group was interpreted as a marker of plasma volume expansion, serum albumin did not differ between groups in the present study.

Urine potassium concentrations were numerically lower in the isotonic group, with a significant difference observed at T12, despite identical potassium concentrations in the isotonic and hypotonic crystalloids (Table 1). This is consistent with relatively lower aldosterone in isotonic group. It is also worth noting that higher urine chloride excretion associated with higher chloride content of isotonic crystalloid sensed by macula densa can suppress aldosterone (36). Given that isotonic crystalloids contain a higher chloride concentration than hypotonic crystalloids, both greater intravascular volume expansion and increased chloride delivery to the macula densa likely contributed to the lower aldosterone concentrations and thus lower urine potassium in isotonic group. The isolated elevation in urine glucose in the hypotonic group at T24 was attributable to a single dog and most likely reflected transient glucosuria from brief exceedance of the renal tubular threshold.

The two crystalloids also differed in their effects on serum electrolytes (Figure 3) and acid–base balance. Serum sodium was lower in the hypotonic group, reaching significance at T24, and individual concentrations fell below the lower reference limit in both phases. The decline was nonetheless modest and self-limiting in healthy Beagles. The largest interval-averaged rate of decline in any dog was 0.58 mEq/L/h over a single 12-h interval, the lowest concentration recorded was 137 mEq/L, hypotonic-phase concentrations recovered toward baseline by T36–T48 while isotonic-phase group means changed little throughout, and no dog showed clinical signs attributable to hyponatremia. Because a comparable downward drift occurred during the isotonic phase, part of this change is likely attributable to factors common to both treatments, fasting and the administration of dextrose-containing maintenance fluid providing free water, rather than to fluid tonicity alone. Serum chloride was higher in the isotonic group at T12 and T24, consistent with its higher chloride content in the fluid after supplementing KCl (Table 1). Correspondingly, the aSID was lower in the isotonic group, although this difference was not statistically significant. The calculated aSID is used to measure the difference between the concentrations of strong cations and strong anions. An increase in aSID leads to metabolic alkalosis, whereas a decrease in aSID indicates acidosis (28). The occasional extreme values observed for serum chloride and the abbreviated strong ion difference were limited to a single dog during the hypotonic phase at T36; no extreme values were identified during the isotonic phase. Excluding these observations and repeating the analyses with non-parametric Wilcoxon signed-rank tests did not change the results or their interpretation. Together with the isolated urine-glucose elevation noted above, these most likely reflect transient individual biological variation rather than a treatment-specific or clinically meaningful effect.

Note that these findings were obtained in healthy Beagle dogs. This apparently intact physiologic integrity of osmoregulatory and volume regulatory mechanisms likely limited the magnitude of the decline in serum sodium despite the large difference in sodium content between the administered fluids. In pediatric patients, concern for hyponatremia primarily arises in the context of hospital-acquired hyponatremia associated with the administration of hypotonic intravenous fluids in the presence of elevated ADH activity (12, 37–39). A similar risk may apply to hospitalized dogs with excessive non-osmotic ADH stimuli in response to factors such as medications, pain, general anesthesia, positive-pressure ventilation, and anxiety (6). Therefore, the findings of the present study should be interpreted as a foundational step toward understanding physiologic responses to fluids of differing tonicity and as a basis for future investigations evaluating the effects of hypotonic crystalloids in hospitalized dogs.

Plasma ADH was lower in the isotonic group at T24 and T48, and several explanations are possible. An osmotic mechanism is unlikely: calculated serum osmolarity was only slightly above the normal canine range at baseline (312 ± 4 vs. 316 ± 4 mOsm/L; *p* = 0.048, small and clinically negligible) (30, 40), declined from baseline in both groups, and remained below the reported canine measured-osmolality threshold for ADH release (>334 mOsm/kg) (32); had osmolarity been the driver, ADH would have been higher, not lower, in the isotonic group. A possible, though unconfirmed, explanation is relative expansion of the effective circulating volume with isotonic crystalloid, suppressing non-osmotic ADH release, with the higher aldosterone in the hypotonic group reflecting greater angiotensin II activity. This must be interpreted with caution: baseline ADH was significantly lower before the isotonic phase (2.5 vs. 7.7 pg/ml; *p* < 0.001) and each group's ADH remained close to its own baseline throughout, so the difference cannot be attributed unambiguously to fluid type and may substantially reflect the baseline imbalance and fixed treatment order. Consistent with this, when ADH was analyzed as change from each phase's own baseline, no between-treatment difference remained (p =0.92; Supplementary Table S4); these findings are therefore consistent with, but do not demonstrate, altered neurohormonal regulation of water balance. Interpretation is further limited by the absence of a validated canine reference interval for this ELISA and the known technical challenges of vasopressin immunoassays. Both phases were assayed identically, so the between-group difference is unlikely to be a measurement artifact.

Both crystalloids were supplemented to achieve the same final potassium concentration of 30 mEq/L, which appeared adequate to prevent sustained potassium depletion during fasting and continuous maintenance fluid administration (3, 30). However, the present study was not designed to evaluate potassium supplementation strategies, and all dogs had apparently normal renal function and intact physiologic regulation. In hospitalized dogs, many factors such as renal dysfunction, oligoanuria, acid–base disturbances, altered aldosterone activity, insulin or catecholamine effects, medications, and ongoing gastrointestinal or urinary losses may substantially alter potassium balance (3, 30). Therefore, potassium supplementation in hospitalized dogs should be individualized and guided by serial electrolyte monitoring.

This study has several limitations. First, because the study was powered for urine output only, the other outcomes were not independently powered and should be interpreted more cautiously. The sample comprised nine healthy Beagles, providing limited statistical power for variables with high inter-individual variability (increased risk of type II error) and limiting generalizability. Although the *a priori* calculation indicated that eight complete paired observations would provide approximately 80% power for the prespecified effect size, 12-h urine-output measurements were missing in two dogs. The complete-case sensitivity analysis therefore included seven dogs and had approximately 75% power under the original assumptions. Although the primary analysis retained all nine dogs using imputation, imputation cannot restore the information lost through missing measurements. Accordingly, the urine-output findings should be interpreted with caution. Second, unlike the MIHMoSA trial (5), treatment order was fixed rather than randomized or counterbalanced. The 10-day washout and baseline-adjusted mixed-effects models were intended to mitigate period and carryover effects, but a fixed sequence cannot fully exclude them. Third, calculated fluid balance was derived from body weight, a practical surrogate that is also influenced by unmeasured insensible and fecal losses. Fourth, these were healthy animals lacking the comorbidities and physiologic derangements that typically prompt maintenance fluid therapy; extrapolation to hospitalized dogs should therefore be cautious. Fifth, beyond carryover, a non-randomized fixed sequence cannot exclude period and order effects on time-dependent physiologic responses. Lastly, although baseline adjustment was applied, baseline serum aldosterone and plasma ADH were substantially higher before the second (hypotonic) phase, so the between-treatment neurohormonal differences may reflect baseline imbalance and/or the fixed treatment order rather than fluid type alone. In addition, serum sodium was measured only at 12-h intervals. Individual rates of change are therefore interval averages, and more rapid transient changes between sampling points cannot be excluded. We also distinguish the absence of concentrations below the reference interval from the absence of a clinically relevant decline from an individual animal's baseline. An animal starting near the upper reference limit may undergo a substantial reduction while remaining within that interval, and this study was not designed to detect it. Because many endpoints and time points were compared without adjustment for multiplicity, and because the overall treatment-by-time interaction for calculated fluid balance was not significant, all timepoint-specific findings, including the T36 fluid-balance difference, should be regarded as exploratory and hypothesis-generating. Finally, the absence of a statistically significant difference for any endpoint should not be interpreted as evidence of equivalence. Instead, it indicates only that no difference was detected under the conditions, sample size, and variability of this experiment. For example, the 48-hour cumulative urine-output ratio was 1.09 (95% CI, 0.78–1.52), so the data remain compatible with effects ranging from a 22% lower to a 52% higher output with the isotonic crystalloid.

## Conclusion

To conclude, in this exploratory study of healthy Beagles, the isotonic and hypotonic maintenance crystalloid formulations produced consistently different renal solute handling. Greater urinary sodium and chloride excretion, higher FENa, more positive cumulative sodium and chloride balance, and more negative electrolyte-free water clearance with the isotonic crystalloid. Whereas the difference in body-weight-based calculated fluid balance was transient and significant only at T36 in exploratory comparisons, and the hypotonic crystalloid did not produce a rapid or clinically important decline in serum sodium over 48 h. Because these findings were obtained in healthy Beagles and the crystalloids differed in more than tonicity, they should be extrapolated to clinical patients with caution, and further studies in hospitalized dogs are warranted.

## Data Availability

The original contributions presented in the study are included in the article/Supplementary material, further inquiries can be directed to the corresponding author.

## References

[1] Beeston D Charnock J Cook S. Current fluid and blood product availability in veterinary setting: a survey of UK small animal practices. J Small Anim Pract. (2020) 61:738–43. doi: 10.1111/jsap.13242 33064320

[2] Hopper K Garcia Rojas A Barter L. An online survey of small animal veterinarians regarding current fluid therapy practices in dogs and cats. J Am Vet Med Assoc. (2018) 252:553–9. doi: 10.2460/javma.252.5.553 29461162

[3] Pardo M Spencer E Odunayo A Ramirez ML Rudloff E Shafford H . 2024 AAHA fluid therapy guidelines for dogs and cats. J Am Anim Hosp Assoc. (2024) 60:131–63. doi: 10.5326/JAAHA-MS-7444 38885492

[4] Waskowski J Salvato SM Müller M Hofer D van Regenmortel N Pfortmueller CA. Choice of creep or maintenance fluid type and their impact on total daily ICU sodium burden in critically ill patients: a systematic review and meta-analysis. J Crit Care. (2023) 78:154403. doi: 10.1016/j.jcrc.2023.154403 37651780

[5] Van Regenmortel N De Weerdt T Van Craenenbroeck AH Roelant E Verbrugghe W Dams K . Effect of isotonic versus hypotonic maintenance fluid therapy on urine output, fluid balance, and electrolyte homeostasis: a crossover study in fasting adult volunteers. Br J Anaesth. (2017) 118:892–900. doi: 10.1093/bja/aex118 28520883 PMC5455256

[6] Hansen B Vigani A. Maintenance fluid therapy: isotonic versus hypotonic solutions. Vet Clin North Am Small Anim Pract. (2017) 47:383–95. doi: 10.1016/j.cvsm.2016.10.001 27908484

[7] Van Regenmortel N Moers L Langer T Roelant E De Weerdt T Caironi P . Fluid-induced harm in the hospital: look beyond volume and start considering sodium. From physiology towards recommendations for daily practice in hospitalized adults. Ann Intensive Care. (2021) 11:79. doi: 10.1186/s13613-021-00851-3 33999276 PMC8128950

[8] McNab S Duke T South M Babl FE Lee KJ Arnup SJ . 140 mmol/L of sodium versus 77 mmol/L of sodium in maintenance intravenous fluid therapy for children in hospital (PIMS): a randomised controlled double-blind trial. Lancet. (2015) 385:1190–7. doi: 10.1016/S0140-6736(14)61459-8 25472864

[9] Alvarez Montañana P Modesto i. Alapont V, Pérez Ocón A, Ortega López P, López Prats JL, Toledo Parreño JD. The use of isotonic fluid as maintenance therapy prevents iatrogenic hyponatremia in pediatrics: a randomized, controlled open study. Pediatr Crit Care Med. (2008) 9:589–97. doi: 10.1097/PCC.0b013e31818d3192 18838929

[10] Neville KA Sandeman DJ Rubinstein A Henry GM McGlynn M Walker JL. Prevention of hyponatremia during maintenance intravenous fluid administration: a prospective randomized study of fluid type versus fluid rate. J Pediatr. (2010) 156:313–319.e2. doi: 10.1016/j.jpeds.2009.07.059 19818450

[11] Moritz ML. Ayus JC. Hypotonic maintenance iv fluids are not appropriate in acutely ill patients Conclusions can't be extrapolated from healthy volunteers. Br J Anaesth. (2017) 119:1064–5. doi: 10.1093/bja/aex376 29077830

[12] Feld LG Neuspiel DR Foster BA Leu MG Garber MD Austin K . Clinical practice guideline: maintenance intravenous fluids in children. Pediatrics. (2018) 142:e20183083. doi: 10.1542/peds.2018-3083 30478247

[13] Van Regenmortel N Verbrugghe W Roelant E Van den Wyngaert T Jorens PG. Maintenance fluid therapy and fluid creep impose more significant fluid, sodium, and chloride burdens than resuscitation fluids in critically ill patients: a retrospective study in a tertiary mixed ICU population. Intensive Care Med. (2018) 44:409–17. doi: 10.1007/s00134-018-5147-3 29589054 PMC5924672

[14] Van Regenmortel N Hendrickx S Roelant E Baar I Dams K Van Vlimmeren K . 154 compared to 54 mmol per liter of sodium in intravenous maintenance fluid therapy for adult patients undergoing major thoracic surgery (TOPMAST): a single-center randomized controlled double-blind trial. Intensive Care Med. (2019) 45:1422–32. doi: 10.1007/s00134-019-05772-1 31576437 PMC6773673

[15] Van Regenmortel N Langer T De Weerdt T Roelant E Malbrain M Van den Wyngaert T . Effect of sodium administration on fluid balance and sodium balance in health and the perioperative setting. Extended summary with additional insights from the MIHMoSA and TOPMAST studies. J Crit Care. (2022) 67:157–65. doi: 10.1016/j.jcrc.2021.10.022 34798374

[16] National Institute for Health and Care Excellence. Intravenous fluid therapy in adults in hospital. Clinical guideline CG174. (2013). Available online at: https://www.nice.org.uk/guidance/cg174 (Accessed March 8, 2026).

[17] Powell-Tuck J Allison SP Gosling P Dileep NL Gordon LC Marcus G . Summary of the British Consensus Guidelines on Intravenous Fluid Therapy for Adult Surgical Patients (GIFTASUP)–for comment. J Intensive Care Soc. (2009) 10:13–5. doi: 10.1177/175114370901000105

[18] National Research Council. Guide for the Care and Use of Laboratory Animals. 8th ed. Washington, DC: National Academies Press (2011).

[19] LabDiet. 5007 - Certified Canine Diet. Available online at: https://www.labdiet.com/product/detail/5007-certified-canine-diet (Accessed May 30, 2026).

[20] Lactated Ringer's Injection USP. Fresenius Kabi USA LLC. (2023). Available online at: https://dailymed.nlm.nih.gov/dailymed/fda/fdaDrugXsl.cfm?setid=8fce1477-991f-4b43-bf05-a80bad897262&type=display (Accessed May 30, 2026).

[21] Dextrose Injection USP (5%). Hospira Inc. (2025). Available online at: https://dailymed.nlm.nih.gov/dailymed/lookup.cfm?setid=10e98cb0-31a5-4ad1-3eaa-aa4af387a42d (Accessed May 30, 2026).

[22] Potassium Chloride for Injection Concentrate USP. Hospira Inc. (2026). Available online at: https://dailymed.nlm.nih.gov/dailymed/drugInfo.cfm?setid=559a0a8c-a8fe-40a5-b196-21f9308780ab (Accessed May 30, 2026).

[23] Normosol-M and 5% Dextrose Injection (Multiple Electrolytes and 5% Dextrose Injection Type 1 USP). ICU Medical Inc. (2020). Available online at: https://dailymed.nlm.nih.gov/dailymed/drugInfo.cfm?setid=aba60c84-94d9-46d0-bdbb-25e0c4e8cc41 (Accessed May 30, 2026).

[24] Villela NR Nascimento Jr P Carvalho LR Teixeira A. Effects of dexmedetomidine on renal system and on vasopressin plasma levels. Experimental study in dogs. Rev Bras Anestesiol. (2005) 55:429–40. doi: 10.1590/S0034-70942005000400007 19468631

[25] Talukder MH Hikasa Y Takahashi H Sato K Matsuu A. Antagonistic effects of atipamezole and yohimbine on medetomidine-induced diuresis in healthy dogs. Can J Vet Res. (2009) 73:260–70. 20046627 PMC2757706

[26] Alves LS Arcoverde KN Oliveira CVA Cavalcante JM Araújo-Silva G Paula VV. Pharmacokinetics and pharmacodynamics of intravenous dexmedetomidine (2 μg/kg) in dogs. Res Vet Sci. (2024) 171:105229. doi: 10.1016/j.rvsc.2024.105229 38507826

[27] Maeckelbergh VA Acierno MJ. Comparison of prothrombin time, activated partial thromboplastin time, and fibrinogen concentration in blood samples collected via an intravenous catheter versus direct venipuncture in dogs. Am J Vet Res. (2008) 69:868–73. doi: 10.2460/ajvr.69.7.868 18593235

[28] Hopper K. Acid-base. Vet Clin North Am Small Anim Pract. (2023) 53:191–206. doi: 10.1016/j.cvsm.2022.07.014 36270834

[29] Arg8-Vasopressin ELISA Kit ADI-900-017A. Enzo Life Sciences Inc. Available online at: https://www.enzo.com/product/arg8-vasopressin-elisa-kit/ (Accessed July 9, 2026).

[30] Wellman ML DiBartola SP Kohn CW. Applied physiology of body fluids in dogs and cats. In:DiBartola SP, ed. Fluid, electrolyte, and acid-base disorders in small animal practice. 4th ed. Philadelphia, PS: Saunders Elsevier (2012) .p. 2–25. doi: 10.1016/B978-1-4377-0654-3.00008-1

[31] Rose BD Post TW. The total body water and the plasma sodium concentration. In: Clinical physiology of acid-base and electrolyte disorders. 5th ed. New York, NY: McGraw-Hill (2001).

[32] Heiene R van Vonderen IK Moe L Mølmen GS Larsen NH Kooistra HS. Vasopressin secretion in response to osmotic stimulation and effects of desmopressin on urinary concentrating capacity in dogs with pyometra. Am J Vet Res. (2004) 65:404–8. doi: 10.2460/ajvr.2004.65.404 15077680

[33] Bie P Sandgaard NCF. Determinants of the natriuresis after acute, slow sodium loading in conscious dogs. Am J Physiol Regul Integr Comp Physiol. (2000) 278:R1–R10. doi: 10.1152/ajpregu.2000.278.1.R1 10644615

[34] Zimmerman RS Edwards BS Schwab TR Heublein DM Burnett JC Jr. Cardiorenal-endocrine dynamics during and following volume expansion. Am J Physiol. (1987) 252:R336–40. doi: 10.1152/ajpregu.1987.252.2.R336 2949632

[35] Melo UP Palhares MS Paes Leme FO Ferreira C Gheller VA Maranhão RPA. Effects of total parenteral nutrition and enteral fluid therapy, with or without glutamine, and crystalloid fluid therapy on the renal function of horses subjected to starvation after exploratory laparotomy. Rev Ciênc Agrovet. (2025) 24:59–75. doi: 10.5965/223811712412025059

[36] Lorenz JN Weihprecht H Schnermann J Skøtt O Briggs JP. Renin release from isolated juxtaglomerular apparatus depends on macula densa chloride transport. Am J Physiol. (1991) 260:F486–93. doi: 10.1152/ajprenal.1991.260.4.F486 2012204

[37] Moritz ML Ayus JC. Prevention of hospital-acquired hyponatremia: a case for using isotonic saline. Pediatrics. (2003) 111:227–30. doi: 10.1542/peds.111.2.227 12563043

[38] Friedman JN. Canadian Paediatric Society, Acute Care Committee. Risk of acute hyponatremia in hospitalized children and youth receiving maintenance intravenous fluids. Paediatr Child Health. (2013) 18:102–4. doi: 10.1093/pch/18.2.102 24421667 PMC3567908

[39] Hall AM Ayus JC Moritz ML. How salty are your fluids? Pediatric maintenance IV fluid prescribing practices among hospitalists. Front Pediatr. (2020) 7:549. doi: 10.3389/fped.2019.00549 32010650 PMC6974532

[40] Dugger DT Epstein SE Hopper K Mellema MS. A comparison of the clinical utility of several published formulae for estimated osmolality of canine serum. J Vet Emerg Crit Care. (2014) 24:188–93. doi: 10.1111/vec.12153 24739033

