# Supplementary material for: Fluid balance, electrolyte, and neurohormonal responses to isotonic vs. hypotonic maintenance crystalloids in healthy Beagle dogs
Source: Front Vet Sci. 2026 Oct 9;13:1948054. doi: 10.3389/fvets.2026.1948054 (PMC13692782; doi:10.3389/fvets.2026.1948054)
Supplement: Supplementary file 1 [file Supplementary_file_1.docx]

**Supplementary Table S1.** Descriptive statistics and baseline-adjusted between-group comparisons over time for all variables in 9 healthy Beagles given isotonic versus hypotonic maintenance crystalloids in two phases described in Table 1.

| **Variable** | **Measure** | **0 h** | **12 h** | **24 h** | **36 h** | **48 h** | **Reference interval** |
| --- | --- | --- | --- | --- | --- | --- | --- |
| **Fluid expansion** |  |  |  |  |  |  |  |
| Cumulative Urine Output (mL) | Isotonic, median (IQR) | 0.0 (0.0–0.0) | 130.0 (100.0–135.0) | 369.0 (277.0–524.0) | 769.0 (670.0–780.0) | 1165.0 (960.0–1246.0) |  |
|  | Hypotonic, median (IQR) | 0.0 (0.0–0.0) | 86.1 (55.0–103.0) | 421.0 (254.0–442.0) | 705.0 (607.0–844.0) | 1111.1 (854.0–1207.1) |  |
|  | Fold change (Iso/Hypo) |  | 1.54 | 1.10 | 1.14 | 1.09 |  |
|  | p value | — | **p = .012** | p = .572 | p = .446 | p = .612 |  |
| Body weight (kg) | Isotonic, mean ± SD | 12.6 ± 1.2 | 12.8 ± 1.2 | 12.8 ± 1.2 | 12.7 ± 1.2 | 12.5 ± 1.1 |  |
|  | Hypotonic, mean ± SD | 12.8 ± 1.1 | 12.9 ± 1.1 | 12.9 ± 1.0 | 12.5 ± 1.1 | 12.7 ± 1.2 |  |
|  | EMM Difference (95% CI) |  | 0.0 (−0.2 to 0.3) | 0.0 (−0.2 to 0.3) | 0.3 (0.1 to 0.6) | 0.0 (−0.3 to 0.2) |  |
|  | p value | p = .213 | p = .863 | p = .693 | **p = .008** | p = .890 |  |
| Urine output (mL) | Isotonic, median (IQR) | 0.0 (0.0–0.0) | 130.0 (100.0–135.0) | 234.0 (160.0–336.0) | 393.0 (295.0–410.0) | 441.0 (290.0–460.0) |  |
|  | Hypotonic, median (IQR) | 0.0 (0.0–0.0) | 95.0 (53.0–106.0) | 318.0 (159.0–382.0) | 351.0 (200.0–402.0) | 364.0 (338.0–479.0) |  |
|  | Fold change (Iso/Hypo) |  | 1.57 | 0.98 | 1.15 | 1.12 |  |
|  | p value | — | p = .065 | p = .915 | p = .534 | p = .619 |  |
| Urine output (ml/kg/h) | Isotonic, median (IQR) | 0.0 (0.0–0.0) | 0.8 (0.7–0.9) | 1.6 (1.1–2.5) | 2.8 (1.7–2.9) | 2.7 (1.8–3.0) |  |
|  | Hypotonic, median (IQR) | 0.0 (0.0–0.0) | 0.6 (0.3–0.8) | 2.2 (0.9–2.4) | 2.2 (1.5–2.5) | 2.3 (2.2–3.1) |  |
|  | Fold change (Iso/Hypo) |  | 1.57 | 0.99 | 1.13 | 1.13 |  |
|  | p value | — | p = .067 | p = .947 | p = .581 | p = .580 |  |
| Calculated fluid balance (mL) | Isotonic, mean ± SD | 0 ± 0 | 222 ± 283 | 222 ± 373 | 61 ± 355 | -111 ± 370 |  |
|  | Hypotonic, mean ± SD | 0 ± 0 | 189 ± 145 | 161 ± 403 | -294 ± 196 | -106 ± 263 |  |
|  | EMM Difference (95% CI) |  | 33 (−217 to 284) | 61 (−189 to 312) | 356 (105 to 606) | −6 (−256 to 245) |  |
|  | p value | — | p = .791 | p = .627 | **p = .006** | p = .965 |  |
| **Volume regulation** |  |  |  |  |  |  |  |
| Calculated urine osmolarity (mOsm/L) | Isotonic, median (IQR) | 1570 (1246–1777) | 1359 (1255–1411) | 512 (403–910) | 448 (434–472) | 417 (409–468) |  |
|  | Hypotonic, median (IQR) | 1288 (1083–1752) | 1409 (1135–1507) | 412 (314–702) | 296 (286–300) | 317 (292–417) |  |
|  | Fold change (Iso/Hypo) |  | 0.99 | 1.52 | 1.53 | 1.40 |  |
|  | p value | p = .803 | p = .918 | **p = .002** | **p = .002** | **p = .011** |  |
| Measured urine osmolality (mOsm/kg) | Isotonic, median (IQR) | 1680 (1245–1720) | 1368 (1325–1533) | 511 (414–894) | 455 (432–466) | 403 (391–472) |  |
|  | Hypotonic, median (IQR) | 1482 (1318–1797) | 1538 (1261–1710) | 452 (355–776) | 330 (298–361) | 313 (310–443) |  |
|  | Fold change (Iso/Hypo) |  | 0.94 | 1.36 | 1.42 | 1.48 |  |
|  | p value | p = .817 | p = .773 | p = .163 | p = .116 | p = .079 |  |
| Aldosterone (pmol/L) | Isotonic, median (IQR) | 51 (48–82) | 75 (48–204) | 31 (15–59) | 46 (36–76) | 29 (8–54) |  |
|  | Hypotonic, median (IQR) | 126 (49–198) | 88 (77–122) | 80 (51–100) | 62 (42–164) | 70 (47–118) |  |
|  | Fold change (Iso/Hypo) |  | 1.07 | 0.46 | 0.80 | 0.41 |  |
|  | p value | p = .023 | p = .845 | **p = .041** | p = .547 | **p = .019** |  |
| Serum Albumin (mg/dL) | Isotonic, mean ± SD | 3.5 ± 0.4 | 3.3 ± 0.3 | 3.3 ± 0.3 | 3.2 ± 0.2 | 3.2 ± 0.3 | 3.3-4.2 |
|  | Hypotonic, mean ± SD | 3.6 ± 0.1 | 3.4 ± 0.1 | 3.3 ± 0.2 | 3.2 ± 0.2 | 3.4 ± 0.1 |  |
|  | EMM Difference (95% CI) |  | 0.0 (−0.2 to 0.1) | 0.0 (−0.1 to 0.2) | 0.1 (−0.1 to 0.3) | −0.1 (−0.3 to 0.0) |  |
|  | p value | p = .314 | p = .712 | p = .628 | p = .184 | p = .077 |  |
| Serum glucose (mg/dL) | Isotonic, median (IQR) | 112 (108–120) | 115 (109–125) | 120 (117–125) | 111 (103–125) | 125 (120–145) | 67-127 |
|  | Hypotonic, median (IQR) | 115 (114–119) | 115 (114–128) | 127 (117–139) | 126 (117–142) | 120 (117–122) |  |
|  | Fold change (Iso/Hypo) |  | 0.96 | 0.90 | 0.98 | 1.07 |  |
|  | p value | p = .699 | p = .647 | p = .278 | p = .819 | p = .469 |  |
| Serum BUN (mg/dL) | Isotonic, median (IQR) | 11 (10–13) | 10 (8–13) | 6 (6–7) | 5 (5–6) | 6 (5–6) | 10-27 |
|  | Hypotonic, median (IQR) | 13 (12–16) | 18 (10–20) | 8 (7–9) | 8 (6–8) | 8 (5–8) |  |
|  | Fold change (Iso/Hypo) |  | 0.74 | 0.89 | 0.82 | 0.87 |  |
|  | p value | p = .226 | p < .001 | p = .168 | **p = .019** | p = .105 |  |
| **Osmoregulation** |  |  |  |  |  |  |  |
| Calculated serum osmolarity (mOsm/L) | Isotonic, mean ± SD | 312 ± 4 | 311 ± 3 | 308 ± 3 | 309 ± 5 | 308 ± 5 |  |
|  | Hypotonic, mean ± SD | 316 ± 4 | 311 ± 7 | 305 ± 3 | 307 ± 3 | 306 ± 3 |  |
|  | EMM Difference (95% CI) |  | 1.1 (−2.5 to 4.6) | 4.5 (1.0 to 8.0) | 2.7 (−0.8 to 6.3) | 2.6 (−0.9 to 6.2) |  |
|  | p value | p = .048 | p = .546 | **p = .013** | p = .126 | p = .142 |  |
| ADH (pg/mL) | Isotonic, mean ± SD | 2.5 ± 1.0 | — | 3.2 ± 1.5 | — | 2.7 ± 1.1 |  |
|  | Hypotonic, mean ± SD | 7.7 ± 3.3 | 9.6 ± 1.9 | 8.5 ± 1.4 | 8.2 ± 1.6 | 7.6 ± 0.9 |  |
|  | EMM Difference (95% CI) |  | NM | −3.8 (−5.5 to −2.1) | NM | −3.4 (−5.1 to −1.7) |  |
|  | p value | p < .001 |  | **p < .001** |  | **p < .001** |  |
| Cumulative EFWC (mL) | Isotonic, mean ± SD | 0 ± 0 | -144 ± 85 | -234 ± 143 | -295 ± 154 | -361 ± 198 |  |
|  | Hypotonic, mean ± SD | 0 ± 0 | -61 ± 43 | 41 ± 126 | 149 ± 162 | 239 ± 199 |  |
|  | EMM Difference (95% CI) |  | -83 (-168 to 3) | -275 (-360 to -190) | -444 (-529 to -359) | -599 (-685 to -514) |  |
|  | p value | — | p = .057 | **p < .001** | p < .001 | p < .001 |  |
| **Serum parameter** |  |  |  |  |  |  |  |
| Serum creatinine (mg/dL) | Isotonic, mean ± SD | 0.5 ± 0.1 | 0.5 ± 0.1 | 0.5 ± 0.1 | 0.6 ± 0.1 | 0.5 ± 0.1 | 0.1-1.3 |
|  | Hypotonic, mean ± SD | 0.7 ± 0.1 | 0.6 ± 0.1 | 0.6 ± 0.1 | 0.6 ± 0.1 | 0.6 ± 0.1 |  |
|  | EMM Difference (95% CI) |  | −0.03 (−0.1 to 0.03) | 0.01 (−0.05 to 0.08) | 0.06 (−0.01 to 0.12) | −0.01 (−0.07 to 0.05) |  |
|  | p value | p = .001 | p = .311 | p = .719 | p = .087 | p = .743 |  |
| Serum sodium (mEq/L) | Isotonic, mean ± SD | 146 ± 1 | 146 ± 1 | 145 ± 2 | 145 ± 2 | 145 ± 3 | 145-154 |
|  | Hypotonic, mean ± SD | 148 ± 2 | 145 ± 2 | 142 ± 2 | 144 ± 2 | 144 ± 2 |  |
|  | EMM Difference (95% CI) |  | 1.7 (0.0 to 3.5) | 3.3 (1.5 to 5.1) | 1.4 (−0.4 to 3.2) | 1.3 (−0.5 to 3.1) |  |
|  | p value | p = .067 | p = .056 | **p = .001** | p = .121 | p = .152 |  |
| Serum potassium (mEq/L) | Isotonic, mean ± SD | 4.4 ± 0.6 | 4.7 ± 0.3 | 4.4 ± 0.3 | 4.9 ± 0.4 | 4.6 ± 0.2 | 3.9-5.5 |
|  | Hypotonic, mean ± SD | 4.4 ± 0.4 | 4.6 ± 0.3 | 4.7 ± 0.5 | 4.6 ± 0.2 | 4.5 ± 0.3 |  |
|  | EMM Difference (95% CI) |  | 0.1 (−0.2 to 0.4) | −0.3 (−0.6 to 0.1) | 0.2 (−0.1 to 0.5) | 0.1 (−0.2 to 0.4) |  |
|  | p value | p = .869 | p = .421 | p = .110 | p = .143 | p = .525 |  |
| Serum chloride (mEq/L) | Isotonic, mean ± SD | 112 ± 1 | 112 ± 1 | 111 ± 2 | 109 ± 2 | 110 ± 3 | 107-118 |
|  | Hypotonic, mean ± SD | 112 ± 2 | 109 ± 2 | 108 ± 2 | 108 ± 5 | 109 ± 1 |  |
|  | EMM Difference (95% CI) |  | 2.5 (0.3 to 4.8) | 2.9 (0.7 to 5.1) | 1.6 (−0.6 to 3.9) | 0.6 (−1.6 to 2.8) |  |
|  | p value | p = .894 | **p = .026** | **p = .012** | p = .150 | p = .604 |  |
| Serum calcium (mg/dL) | Isotonic, mean ± SD | 10.7 ± 0.7 | 9.5 ± 0.9 | 10.3 ± 0.5 | 9.6 ± 1.1 | 10.3 ± 0.4 | 9.1-11.5 |
|  | Hypotonic, mean ± SD | 10.5 ± 0.8 | 10.3 ± 0.4 | 10.3 ± 0.2 | 9.8 ± 1.2 | 10.8 ± 0.5 |  |
|  | EMM Difference (95% CI) |  | −0.9 (−1.4 to −0.3) | 0.0 (−0.6 to 0.6) | −0.3 (−0.9 to 0.3) | −0.5 (−1.1 to 0.0) |  |
|  | p value | p = .675 | **p = .004** | p = .972 | p = .274 | p = .058 |  |
| Serum phosphorous (mg/dL) | Isotonic, mean ± SD | 4.4 ± 1.0 | 4.0 ± 0.8 | 3.9 ± 0.6 | 4.0 ± 0.6 | 4.2 ± 0.6 | 2.2-6.3 |
|  | Hypotonic, mean ± SD | 4.5 ± 0.8 | 4.4 ± 1.2 | 4.0 ± 0.6 | 4.2 ± 0.9 | 4.3 ± 0.7 |  |
|  | EMM Difference (95% CI) |  | −0.3 (−0.8 to 0.2) | 0.0 (−0.5 to 0.5) | −0.2 (−0.7 to 0.3) | 0.0 (−0.5 to 0.5) |  |
|  | p value | p = .532 | p = .247 | p = .855 | p = .447 | p = .855 |  |
| Abbreviated Strong Ion Difference | Isotonic, mean ± SD | 38.7 ± 1.4 | 38.7 ± 1.2 | 38.2 ± 1.9 | 40.8 ± 1.9 | 39.8 ± 1.6 |  |
|  | Hypotonic, mean ± SD | 40.2 ± 1.5 | 39.8 ± 2.4 | 38.6 ± 1.6 | 41.3 ± 6.0 | 39.4 ± 1.3 |  |
|  | EMM Difference (95% CI) |  | −1.3 (−3.8 to 1.3) | −0.1 (−2.6 to 2.5) | −0.7 (−3.2 to 1.9) | 0.2 (−2.3 to 2.8) |  |
|  | p value | p = .215 | p = .318 | p = .951 | p = .589 | p = .848 |  |
| Serum TCO2 (mEq/L) | Isotonic, mean ± SD | 22.0 ± 1.5 | 23.0 ± 1.5 | 22.7 ± 1.9 | 24.6 ± 2.2 | 24.1 ± 2.1 | 18-27 |
|  | Hypotonic, mean ± SD | 22.9 ± 1.9 | 24.2 ± 2.4 | 23.4 ± 1.3 | 22.7 ± 2.3 | 23.9 ± 1.6 |  |
|  | Between-group |  | −0.8 (−2.4 to 0.7); −0.3 (−1.9 to 1.2); 2.3 (0.7 to 3.8); 0.6 (−1.0 to 2.2) |  |  |  |  |
|  | p value | p = .267 | p = .297 | p = .671 | p = .005 | p = .453 |  |
| **Urine parameter** |  |  |  |  |  |  |  |
| Urine sodium (mEq/L) | Isotonic, mean ± SD | 81.1 ± 28.0 | 203.0 ± 45.5 | 165.9 ± 51.7 | 141.4 ± 24.4 | 129.6 ± 18.1 |  |
|  | Hypotonic, mean ± SD | 74.8 ± 34.1 | 88.2 ± 49.0 | 51.4 ± 23.5 | 49.6 ± 16.8 | 62.2 ± 15.9 |  |
|  | EMM Difference (95% CI) |  | 112.5 (82.5 to 142.6) | 117.3 (87.3 to 147.3) | 92.5 (62.5 to 122.5) | 71.2 (41.2 to 101.3) |  |
|  | p value | p = .689 | **p < .001** | **p < .001** | **p < .001** | **p < .001** |  |
| Urine potassium (mEq/L) | Isotonic, mean ± SD | 114.8 ± 54.8 | 95.2 ± 45.0 | 46.2 ± 29.2 | 34.2 ± 16.8 | 42.9 ± 20.1 |  |
|  | Hypotonic, mean ± SD | 134.0 ± 60.6 | 157.5 ± 40.5 | 57.7 ± 29.2 | 48.5 ± 33.8 | 47.4 ± 16.6 |  |
|  | EMM Difference (95% CI) |  | −54.2 (−78.5 to −30.0) | −8.7 (−32.9 to 15.6) | −2.9 (−27.1 to 21.4) | 0.1 (−24.2 to 24.3) |  |
|  | p value | p = .178 | **p < .001** | p = .476 | p = .813 | p = .996 |  |
| Urine chloride (mEq/L) | Isotonic, mean ± SD | 64.7 ± 52.9 | 233.0 ± 63.2 | 181.3 ± 85.1 | 145.8 ± 34.5 | 137.2 ± 25.2 |  |
|  | Hypotonic, mean ± SD | 131.4 ± 92.3 | 189.0 ± 92.4 | 101.5 ± 55.0 | 66.2 ± 19.5 | 75.6 ± 20.5 |  |
|  | EMM Difference (95% CI) |  | 79.0 (28.3 to 129.7) | 105.8 (55.1 to 156.5) | 103.8 (53.1 to 154.5) | 89.0 (38.3 to 139.7) |  |
|  | p value | **p = .012** | **p = .003** | **p < .001** | **p < .01** | **p = .01** |  |
| Urine glucose (mg/dL) | Isotonic, median (IQR) | 20 (13–27) | 19 (14–21) | 5 (4–9) | 4 (3–6) | 3 (3–4) |  |
|  | Hypotonic, median (IQR) | 30 (26–34) | 26 (17–28) | 12 (5–33) | 5 (3–9) | 4 (3–6) |  |
|  | Fold change (Iso/Hypo) |  | 0.79 | 0.41 | 0.76 | 0.91 |  |
|  | p value | p = .231 | p = .591 | **p = .046** | p = .528 | p = .834 |  |
| Urine BUN (mg/dL) | Isotonic, median (IQR) | 3105 (2396–3780) | 2155 (1875–2289) | 408 (338–1015) | 305 (279–347) | 301 (255–337) |  |
|  | Hypotonic, median (IQR) | 2628 (2134–3514) | 2387 (1745–2610) | 663 (406–1101) | 365 (294–453) | 328 (283–432) |  |
|  | Fold change (Iso/Hypo) |  | 0.86 | 0.99 | 0.87 | 0.93 |  |
|  | p value | p = .666 | p = .399 | p = .952 | p = .436 | p = .685 |  |
| Urine creatinine (mg/dL) | Isotonic, median (IQR) | 302.8 (206.9–396.5) | 214.7 (169.0–275.4) | 61.3 (53.2–92.7) | 51.5 (50.0–63.5) | 53.8 (46.7–61.6) |  |
|  | Hypotonic, median (IQR) | 300.7 (269.1–309.9) | 204.0 (158.5–269.8) | 57.7 (53.0–78.9) | 50.1 (42.5–59.9) | 58.7 (47.0–64.2) |  |
|  | Fold change (Iso/Hypo) |  | 1.01 (0.73–1.39) | 1.20 (0.87–1.65) | 1.04 (0.75–1.44) | 1.07 (0.78–1.48) |  |
|  | p value | p = .773 | p = .961 | p = .269 | p = .806 | p = .661 |  |
| FENa (%) | Isotonic, median (IQR) | 0.1 (0.1–0.2) | 0.3 (0.3–0.4) | 0.9 (0.7–0.9) | 1.1 (0.7–1.3) | 0.9 (0.7–0.9) |  |
|  | Hypotonic, median (IQR) | 0.1 (0.1–0.2) | 0.2 (0.1–0.2) | 0.3 (0.3–0.4) | 0.4 (0.3–0.5) | 0.5 (0.4–0.7) |  |
|  | Fold change (Iso/Hypo) |  | 2.13 | 2.64 | 2.76 | 1.71 |  |
|  | p value | **p = .001** | p < .001 | **p < .001** | p < .001 | **p = .010** |  |

Values in the first two rows are mean ± SD for endpoints analyzed on the original scale and median (interquartile range, 25th–75th percentile) for log-transformed endpoints (those reported with a fold change). Values in the third row are EMM Difference = difference in estimated marginal means, adjusted for baseline and missing values or; Fold Change = ratio of back-transformed EMM (i.e. geometric mean ratio)) a positive difference indicates isotonic > hypotonic and a negative difference indicates hypotonic > isotonic. Fold change > 1 indicates isotonic > hypotonic. NM = not measured; – = not applicable. Significant p values (< .05) are bold.

aSID = abbreviated strong ion difference; ADH = antidiuretic hormone; BUN = blood urea nitrogen; CI = confidence interval; EFWC = electrolyte-free water clearance; FENa = fractional excretion of sodium; SD = standard deviation; TCO2 = total carbon dioxide.

**Supplementary Table S2. Individual serum sodium concentrations (mEq/L) in 9 healthy Beagles during isotonic and hypotonic maintenance crystalloid administration.**

**Isotonic phase (lactated Ringer’s solution + 5% dextrose + 26 mEq/L KCl)**

| **Dog** | **T0** | **T12** | **T24** | **T36** | **T48** | **Most negative 12-h change** | **Most negative change from baseline** | **Nadir** |
| --- | --- | --- | --- | --- | --- | --- | --- | --- |
| 1 | 146 | 145 | 142 | 142 | 141 | −3 | −5 | 141 |
| 2 | 146 | 146 | 145 | 146 | 144 | −2 | −2 | 144 |
| 3 | 146 | 148 | 144 | 147 | 144 | −4 | −2 | 144 |
| 4 | 145 | 144 | 147 | 144 | 142 | −3 | −3 | 142 |
| 5 | 146 | 145 | 147 | 144 | 145 | −3 | −2 | 144 |
| 6 | 145 | 146 | 144 | 143 | 143 | −2 | −2 | 143 |
| 7 | 148 | 146 | 144 | 148 | 148 | −2 | −4 | 144 |
| 8 | 146 | 146 | 147 | 147 | 150 | 0 | 0 | 146 |
| 9 | 149 | 147 | 144 | 146 | 146 | −3 | −5 | 144 |

**Hypotonic phase (Normosol-M + 5% dextrose + 17 mEq/L KCl)**

| **Dog** | **T0** | **T12** | **T24** | **T36** | **T48** | **Most negative 12-h change** | **Most negative change from baseline** | **Nadir** |
| --- | --- | --- | --- | --- | --- | --- | --- | --- |
| 1 | 146 | 144 | 142 | 143 | 144 | −2 | −4 | 142 |
| 2 | 145 | 144 | 137 | 145 | 143 | −7 | −8 | 137 |
| 3 | 146 | 148 | 142 | 145 | 145 | −6 | −4 | 142 |
| 4 | 151 | 147 | 143 | 145 | 142 | −4 | −9 | 142 |
| 5 | 148 | 146 | 143 | 144 | 143 | −3 | −5 | 143 |
| 6 | 147 | 145 | 141 | 144 | 142 | −4 | −6 | 141 |
| 7 | 149 | 142 | 145 | 144 | 145 | −7 | −7 | 142 |
| 8 | 151 | 144 | 142 | 147 | 147 | −7 | −9 | 142 |
| 9 | 148 | 141 | 143 | 141 | 144 | −7 | −7 | 141 |

Reference interval, 145–154 mEq/L. Negative values indicate a decrease, consistent with Figure 4C; 0 indicates no decrease over any interval. Because serum sodium was measured every 12 hours, per-interval changes are interval averages and cannot exclude more rapid transient changes between sampling points. Summary: most negative single-interval change, −4 (isotonic) vs −7 (hypotonic) mEq/L; most negative change from an individual baseline, −5 vs −9 mEq/L; dogs with any 12-h decline exceeding 6 mEq/L (>0.5 mEq/L/h), 0/9 vs 4/9; lowest recorded concentration, 141 vs 137 mEq/L; postbaseline values <145 mEq/L, 15/36 (8/9 dogs) vs 24/36 (9/9 dogs).

**Supplementary Table S3. Overall (Type 3) tests of fixed effects (treatment, time, and treatment-by-time interaction) from the linear mixed-effects models for each endpoint.**

| **Endpoint** | **Treatment (p value)** | **Time**  **(p value)** | **Treatment × time (p value)** |
| --- | --- | --- | --- |
| Calculated fluid balance (mL) | .081 | <.001 | .17 |
| Fluid balance, intake − output (mL) | .19 | .003 | .37 |
| Body weight (kg) | .12 | <.001 | .16 |
| Cumulative urine output (mL)* | .028 | <.001 | .41 |
| Urine output (mL/kg/h)* | .12 | <.001 | .53 |
| Cumulative EFWC (mL) | <.001 | .49 | <.001 |
| FENa (%)* | <.001 | <.001 | .32 |
| Urine sodium (mEq/L) | <.001 | <.001 | .073 |
| Urine chloride (mEq/L) | <.001 | <.001 | .69 |
| Urine potassium (mEq/L) | <.001 | <.001 | .007 |
| Serum sodium (mEq/L) | <.001 | .035 | .33 |
| Serum chloride (mEq/L) | .001 | .042 | .46 |
| Serum potassium (mEq/L) | .51 | .16 | .15 |
| Serum calcium (mg/dL) | .004 | <.001 | .19 |
| Serum albumin (g/dL) | .87 | .084 | .16 |
| Serum BUN (mg/dL)* | <.001 | <.001 | .34 |
| Calculated serum osmolarity (mOsm/L) | .013 | .004 | .76 |
| Calculated urine osmolarity (mOsm/L)* | <.001 | <.001 | .10 |
| Measured urine osmolality (mOsm/kg)* | .056 | <.001 | .49 |
| Aldosterone (pmol/L)* | .035 | .068 | .18 |
| ADH (pg/mL) | <.001 | .042 | .67 |

Models as in the Methods: fixed effects for treatment, time, and treatment × time, baseline covariate where a non-zero baseline was present, random intercept per dog; restricted maximum likelihood with Satterthwaite degrees of freedom. *Log-transformed. ADH compared at T24 and T48 only. EFWC = electrolyte-free water clearance; FENa = fractional excretion of sodium; ADH = antidiuretic hormone.

**Supplementary Table S4. Supplementary and sensitivity analyses.**

**(A) Fluid balance calculated as cumulative infused volume minus cumulative urine output (isotonic − hypotonic).**

| **Time** | **EMM difference (mL)** | **95% CI** | **p value** |
| --- | --- | --- | --- |
| T24 | +4 | −170 to 177 | .97 |
| T36 | −185 | −397 to 27 | .086 |
| T48 | −31 | −205 to 142 | .72 |

Not calculable at T12 of the isotonic phase (infused volumes not recorded for that interval); intervals with imputed urine volumes excluded.

**(B) Cumulative sodium and chloride balance, intake minus urinary excretion (isotonic − hypotonic).**

| **Endpoint** | **T24** | **T36** | **T48** |
| --- | --- | --- | --- |
| Na balance (mEq) | +31.9 (p=.001) | +28.0 (p=.011) | +51.5 (p<.001) |
| Cl balance (mEq) | +36.7 (p=.001) | +35.0 (p=.010) | +52.2 (p<.001) |

**(C) Cumulative urine output under three missing-data approaches (fold change, isotonic/hypotonic; p value).**

| **Approach** | **T12** | **T24** | **T36** | **T48** |
| --- | --- | --- | --- | --- |
| Group-mean imputation (primary) | 1.54 (.012) | 1.10 (.57) | 1.14 (.45) | 1.09 (.61) |
| Excluding 2 affected dogs (n = 7) | 1.58 (.014) | 1.25 (.22) | 1.22 (.27) | 1.14 (.48) |
| Values left missing (REML) | 1.58 (.014) | 1.25 (.22) | 1.22 (.26) | 1.14 (.48) |

**(D) Change-from-baseline sensitivity analysis for the five variables with baseline differences between phases (isotonic − hypotonic difference in change from own phase baseline).**

| **Endpoint** | **Overall treatment**  **(p value)** | **T12** | **T24** | **T36** | **T48** |
| --- | --- | --- | --- | --- | --- |
| ADH (pg/mL) | .92 | — | −0.1 (.90) | — | +0.3 (.79) |
| Aldosterone (fold)* | .93 | 1.71 (.13) | 0.74 (.39) | 1.28 (.48) | 0.66 (.23) |
| Serum creatinine (mg/dL) | <.001 | +0.02 (.50) | +0.07 (.045) | +0.11 (.001) | +0.04 (.18) |
| Urine chloride (mEq/L) | <.001 | +111 (<.001) | +147 (<.001) | +146 (<.001) | +128 (<.001) |
| Calc serum osmolarity (mOsm/L) | <.001 | +4.2 (.067) | +7.0 (.003) | +4.6 (.049) | +5.7 (.015) |

*Log scale. Paired Wilcoxon signed-rank tests at each time point were concordant. ADH available in 6 dogs at T24 and T48.
